# Supplementary figures and images for: Day-night and seasonal variation of human gene expression across tissues
Source: PLoS Biol. 2023 Feb 6;21(2):e3001986. doi: 10.1371/journal.pbio.3001986 (PMC9934459; doi:10.1371/journal.pbio.3001986)

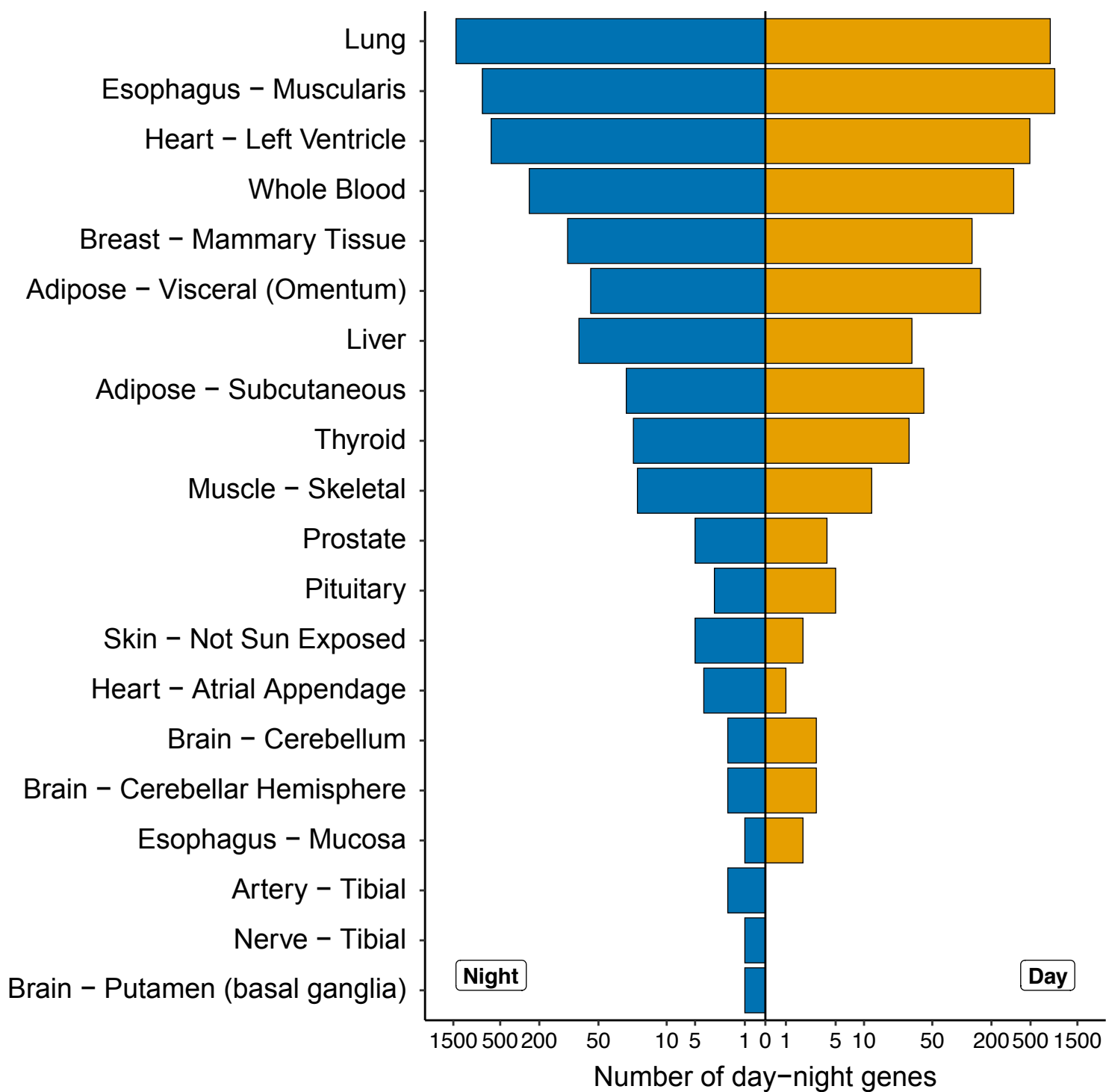

Supplement: S1 Fig — Number of genes found as day-night, i.e., genes differentially expressed between day and night for each tissue using FDR ≤ 0.1. Tissues are sorted by the total number of day-night genes. The data underlying this figure can be found in S1 Data. (PDF) [file pbio.3001986.s001.pdf]

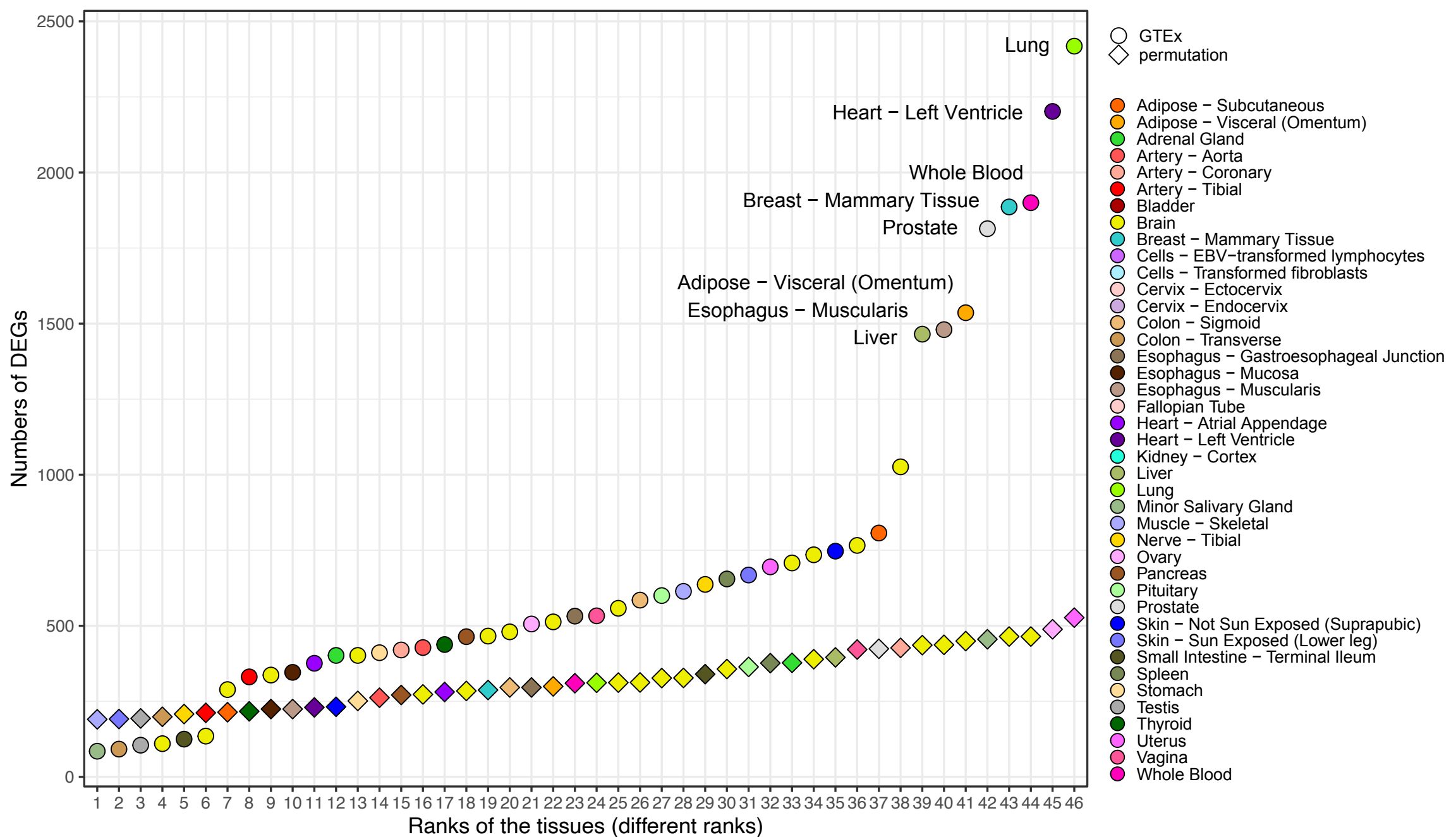

Supplement: S2 Fig — For each tissue, the number of day-night genes from the real data (GTEx, circle) or the median number of day-night genes over the 1,000 random permutations (permutation, diamond) was computed (y-axis). The tissues were ordered according their number of day-night genes (x-axis), independently for the GTEx and the permutation dataset. DEGs, differentially expressed genes. The data underlying this figure can be found in S1 Data. (PDF) [file pbio.3001986.s002.pdf]

**A**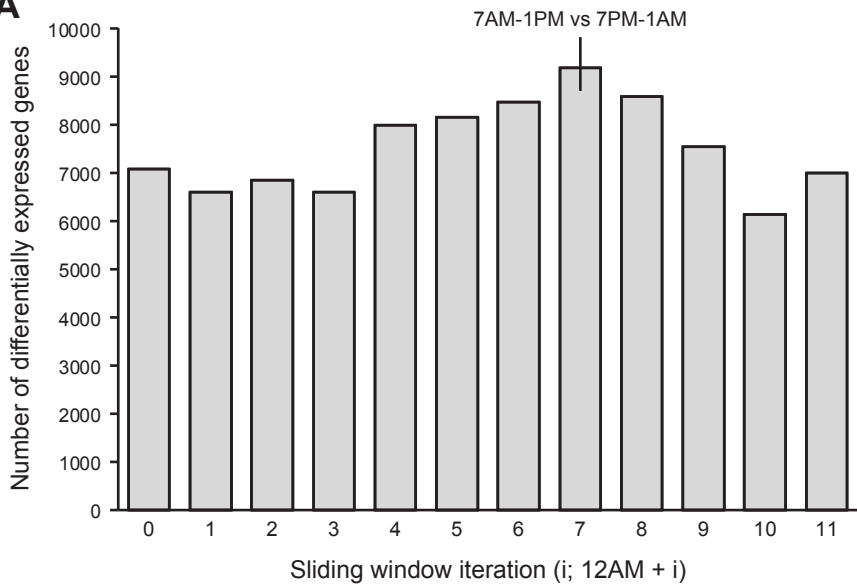**B**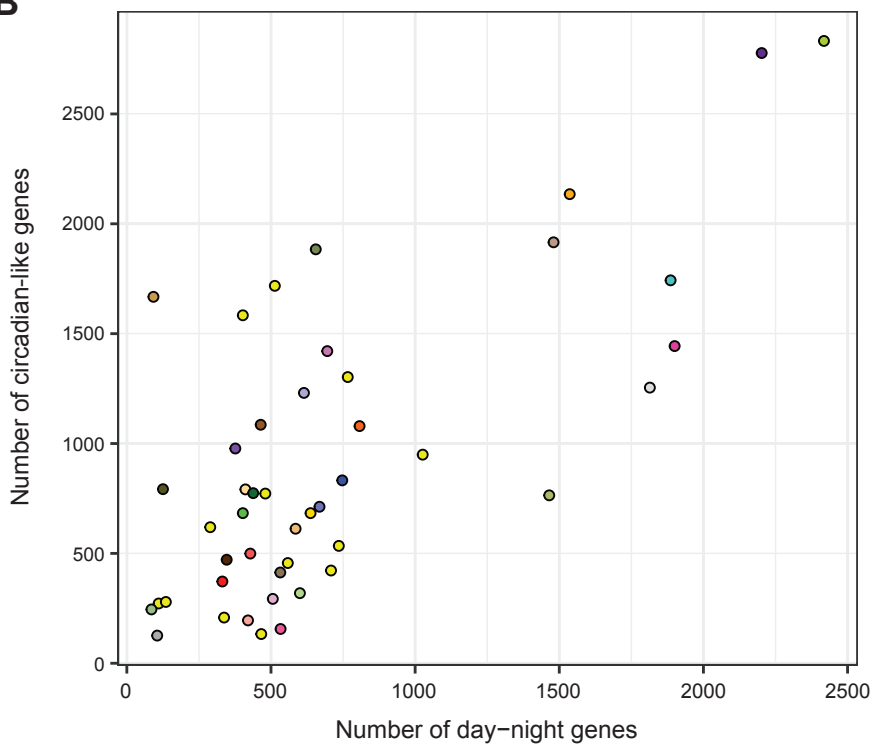

Supplement: S3 Fig — (A) Number of differentially expressed genes between sliding pairs of 6-hour time windows, defined as [i,i+6) and [i+12,i+18) for i = 0…11. Each step i corresponds to 1 hour. (B) Scatter plot showing the number of genes that are differentially expressed between at least one pair of time windows (“circadian-like” genes) and of day-night genes per tissue. The data underlying this figure can be found in S1 Data. (PDF) [file pbio.3001986.s003.pdf]

Absolute log2 fold-change

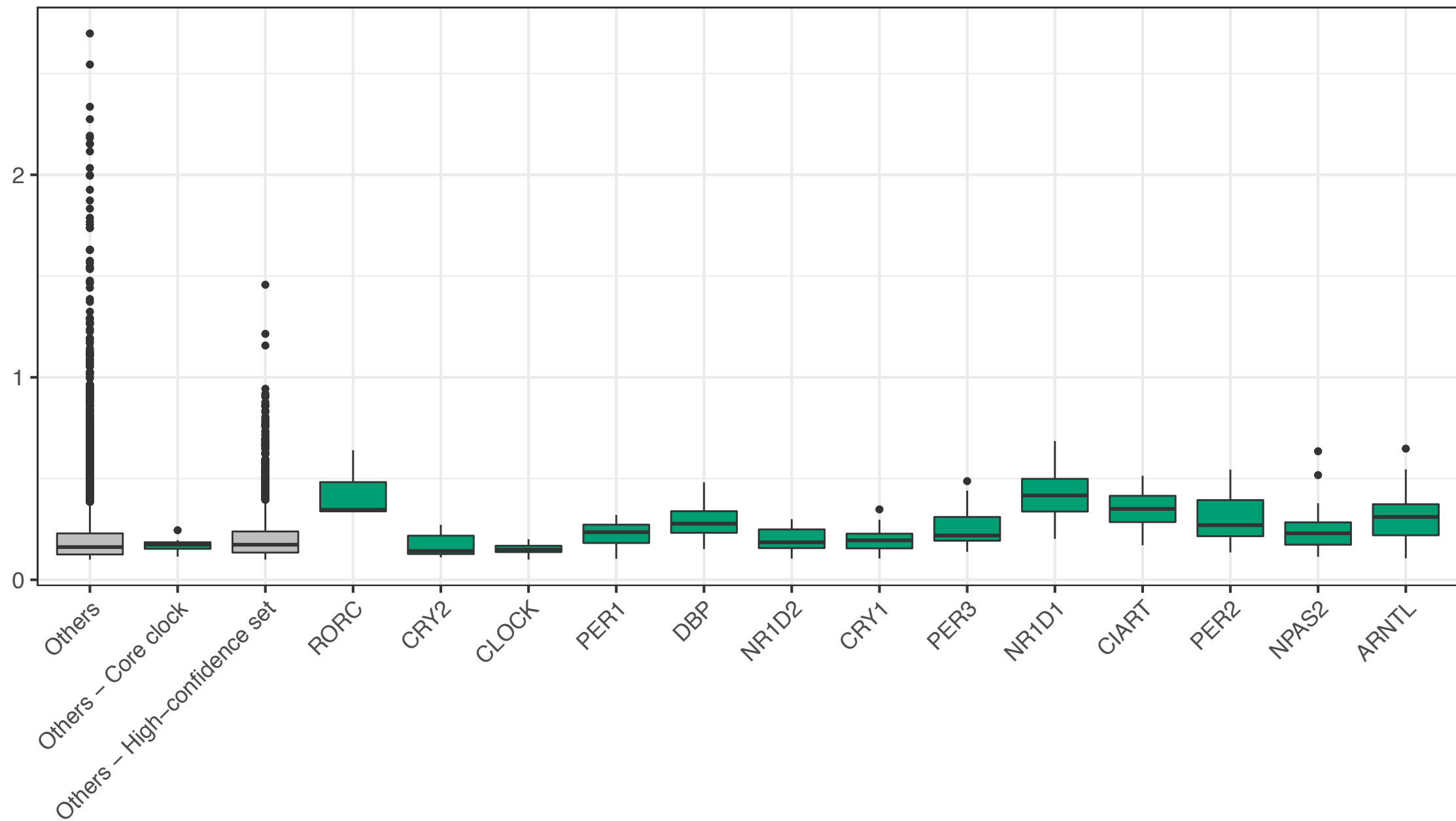

Supplement: S4 Fig — Each core clock gene in the high-confidence day-night set is plotted separately (green boxplots). The rest of the genes are pooled together in “Others—Core clock” if they are in the list of the core clock genes, in “Others—High-confidence set” if they are part of the high-confidence set, or in “Others” otherwise. The data underlying this figure can be found in Supp Dataset 1 (https://doi.org/10.6084/m9.figshare.21906252.v1). (PDF) [file pbio.3001986.s004.pdf]

TPM

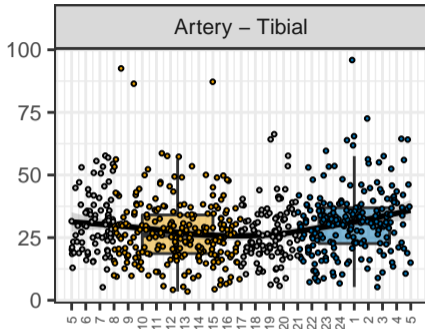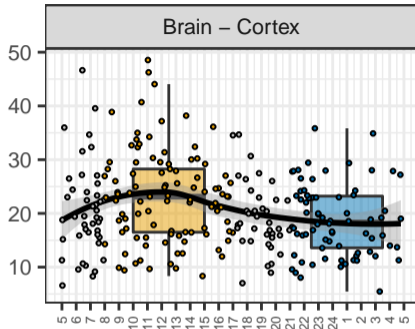

Classification 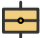 Day 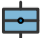 Night 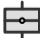 Twilight

Supplement: S5 Fig — TPM values for NR1D2 in the artery—tibial (left) and in the brain—cortex (right). The colors of the dots represent the classification of the individuals according to the time of death of the donor: during the day (yellow), during the night (blue), or in-between for twilight (grey). The samples classified as twilight have been discarded for the day-night analysis. The “circadian” curve was created using the geom_smooth function from ggplot2 in R with the “loess” method. The data underlying this figure can be found in S1 Data. (PDF) [file pbio.3001986.s005.pdf]

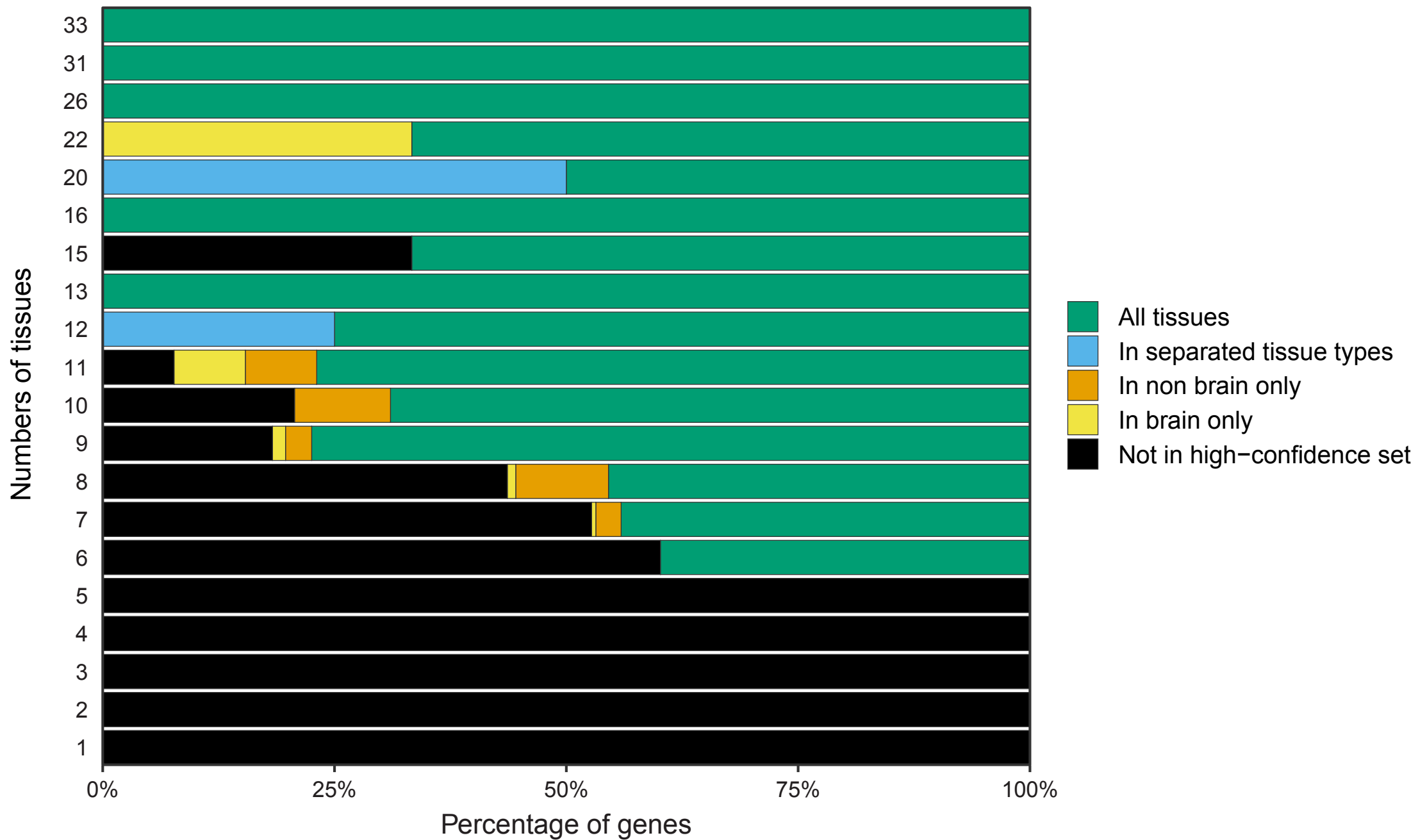

Supplement: S6 Fig — For each number of tissues in which a gene was identified as day-night, the percentage of genes that are consistently up-regulated either during day or during night for (i) all tissues taken together (P ≤ 0.05 for the binomial test on all tissues; All tissues, green); (ii) in non-brain and in brain tissues separately (P ≤ 0.05 for the binomial test on non-brain and brain tissues and P > 0.05 for all tissues; In separated tissue types, blue); (iii) in non-brain tissues only (P ≤ 0.05 only for the non-brain tissues test; In non-brain only, orange); (iv) in brain tissues only (P ≤ 0.05 only for the brain tissues test; In brain only, yellow); and (v) inconsistent between day and night tissues (P > 0.05 for all binomial tests; Not in high-confidence set, black). See Methods for details. The data underlying this figure can be found in S8 Table. (PDF) [file pbio.3001986.s006.pdf]

**A*****THRA* – Thyroid**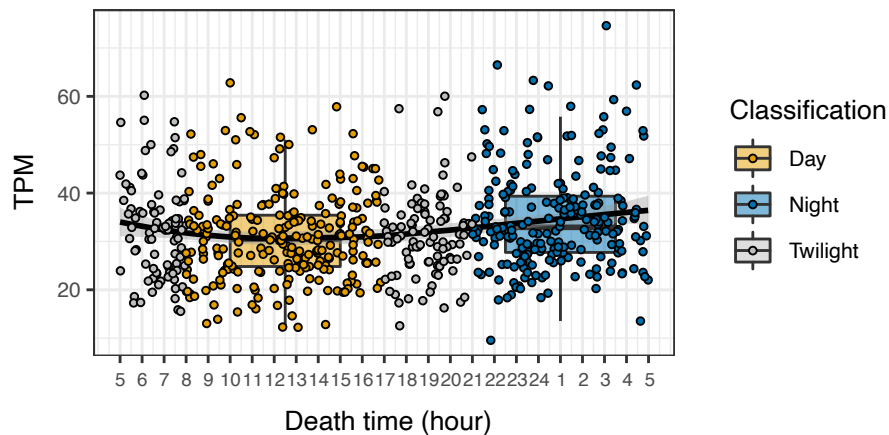**C*****NIPB5* – Esophagus – Gastroesoph. J.**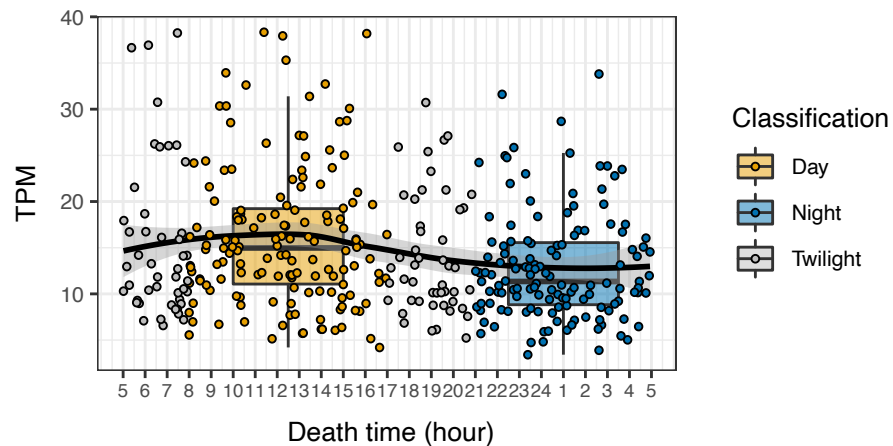**B*****RPS26* – Whole Blood**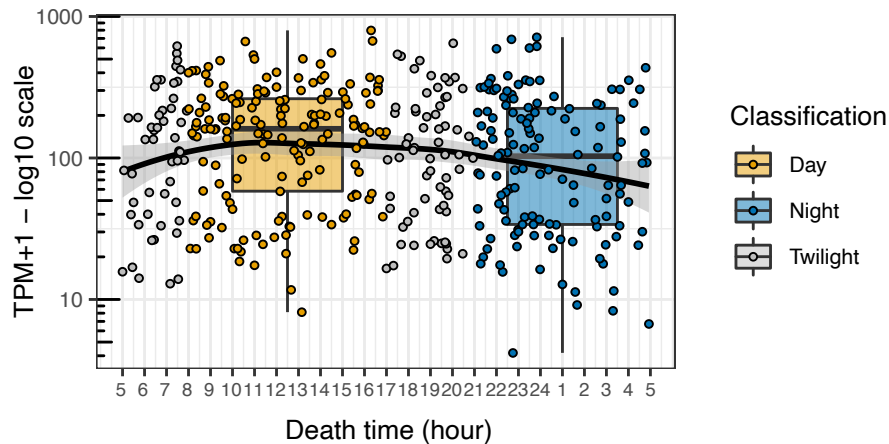**D*****TRIM22* – Adipose – Visceral**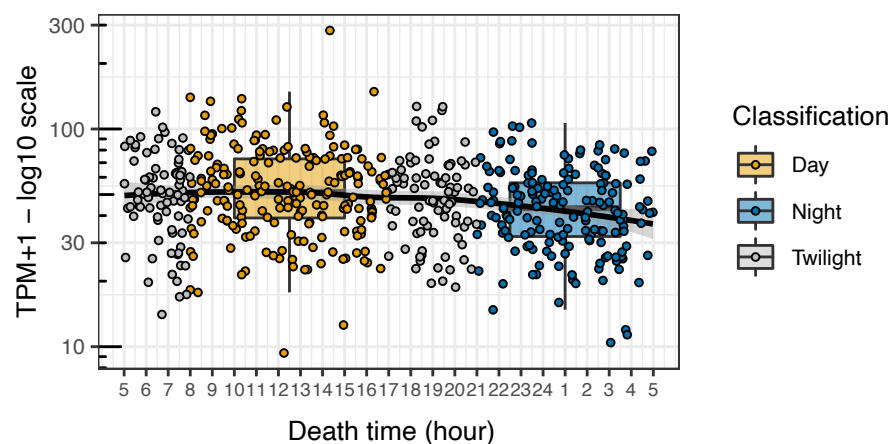

Supplement: S7 Fig — Expression values (TPM) values for three high-confidence day-night genes at the time of death of the GTEx donors: (A) THRA in thyroid, (B) RPS26 in the whole blood, (C) NPIPB5 in the esophagus-gastroesophageal junction, and (D) TRIM22 in the adipose-visceral (omentum). The colors of the dots represent the classification of the individuals according to their time of death of the donor: during the day (yellow), during the night (blue), or in-between for twilight (grey). The samples classified as twilight have been discarded for the day-night analysis. The “circadian” curve was created using the geom_smooth function from ggplot2 in R with the “loess” method. The data underlying this figure can be found in S1 Data. (PDF) [file pbio.3001986.s007.pdf]

Absolute logFC

2

1

0

Day-night

Spring

Summer

Fall

Winter

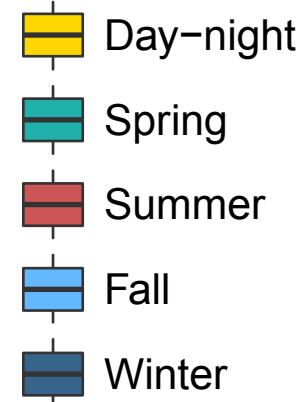

Supplement: S10 Fig — Boxplots showing the absolute log2 fold-change (or effect size) of day-night genes (yellow) or seasonal genes (red: summer, green: spring, light blue: fall, dark blue: winter). The data underlying this figure can be found in Supp Datasets 1 and 2 (https://doi.org/10.6084/m9.figshare.21906252.v1 and https://doi.org/10.6084/m9.figshare.21906255.v1, respectively). (PDF) [file pbio.3001986.s010.pdf]

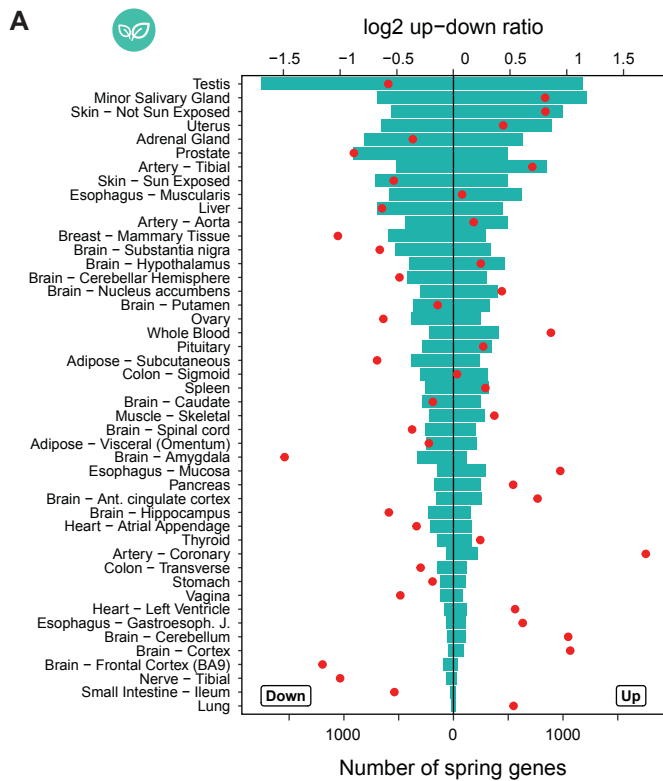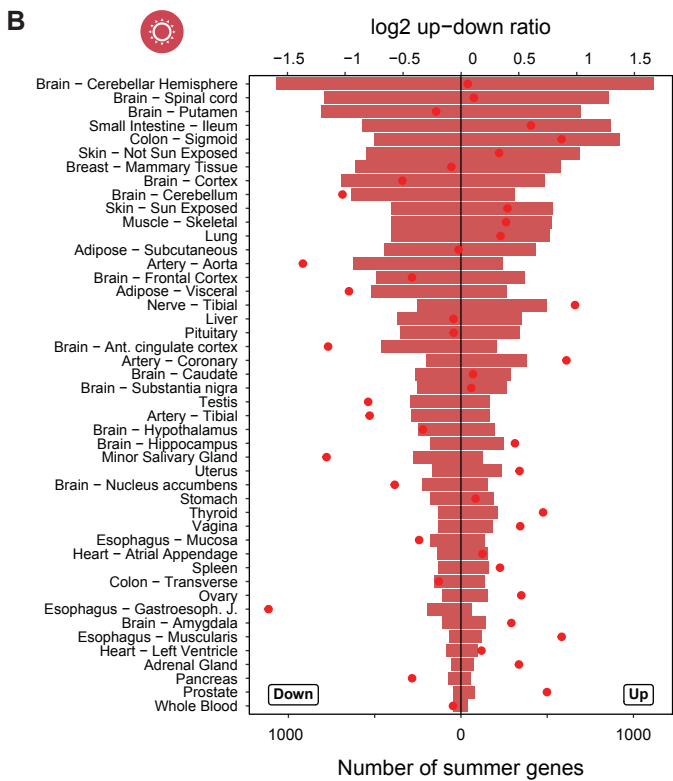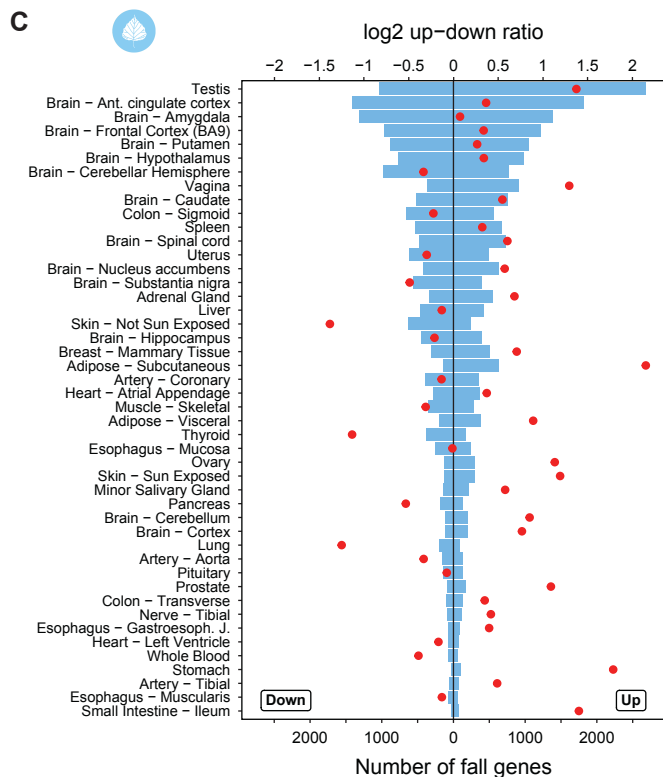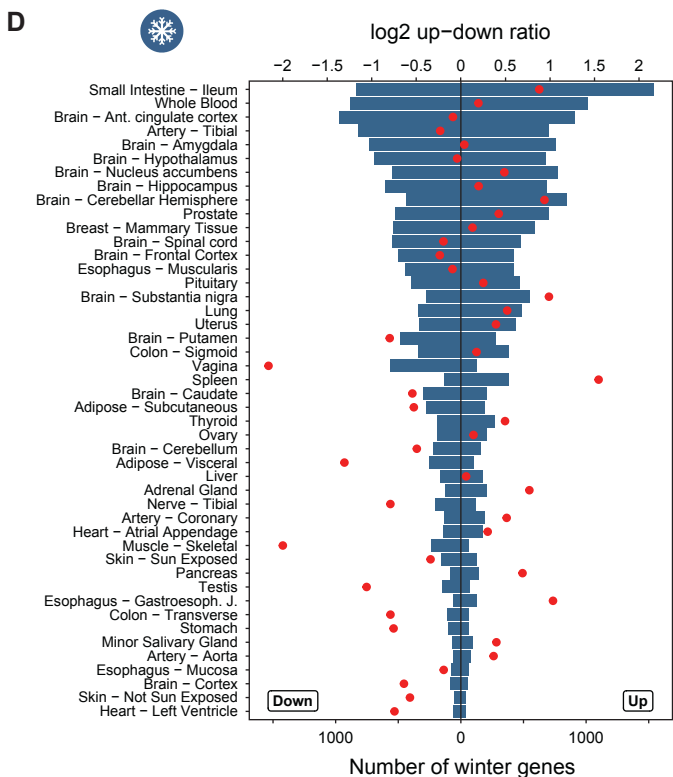

Supplement: S11 Fig — Number of overexpressed (Season up, right side) and underexpressed (Season down, left side) seasonal genes per tissue in GTEx (bottom axis) for each season, spring (A), summer (B), fall (C), and winter (D). The tissues were ordered according to the total number of seasonal genes per season. The red dot represents the log2 ratio between the number of over and under genes (top axis). The data underlying this figure can be found in S1 Data. (PDF) [file pbio.3001986.s011.pdf]

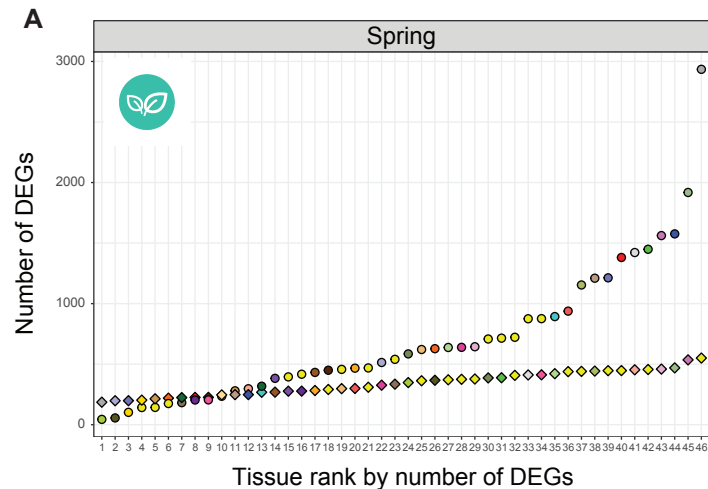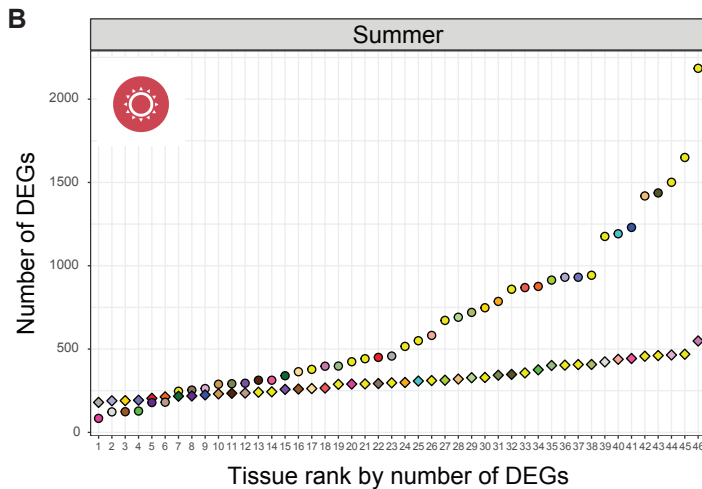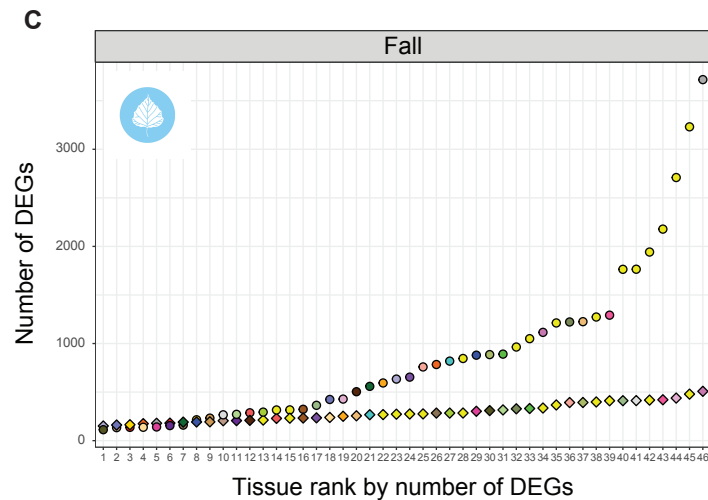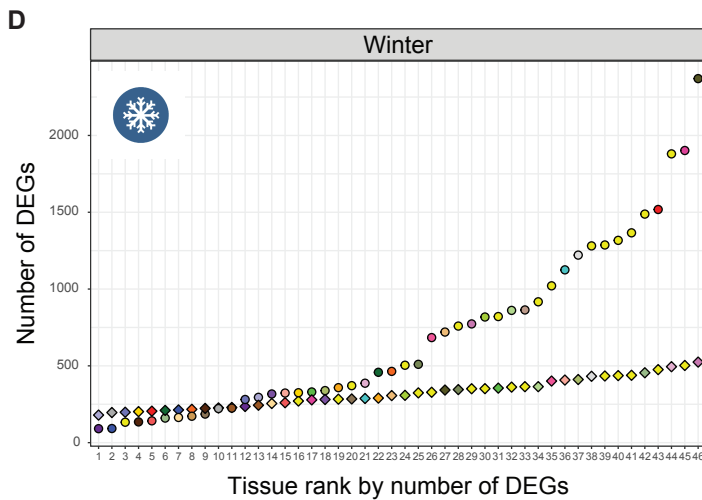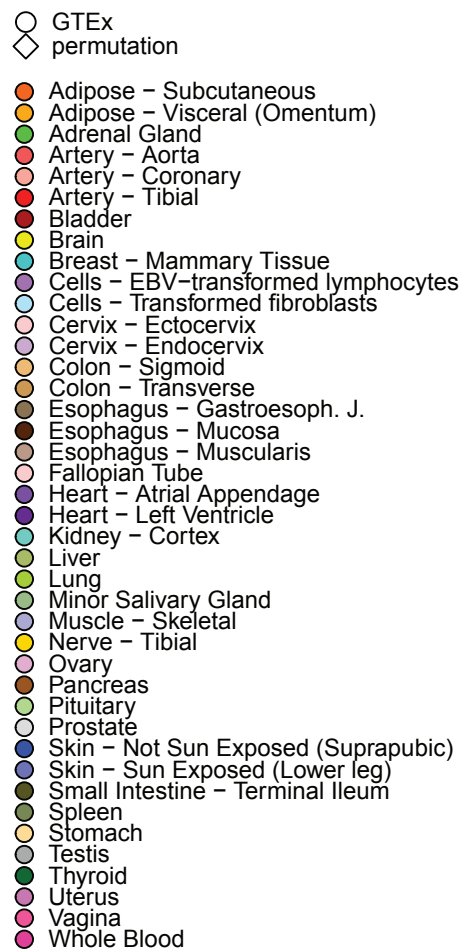

Supplement: S12 Fig — For each tissue, the number of spring (A), summer (B), fall (C), or winter (D) genes from the real data (GTEx, circle) or the median number of genes differentially expressed in each season over the 1,000 random permutations (permutation, diamond) was computed (y-axis). The tissues were ordered according their number of seasonal genes (x-axis), independently for the GTEx and the permutation dataset. DEGs, differentially expressed genes. The data underlying this figure can be found in S1 Data. (PDF) [file pbio.3001986.s012.pdf]

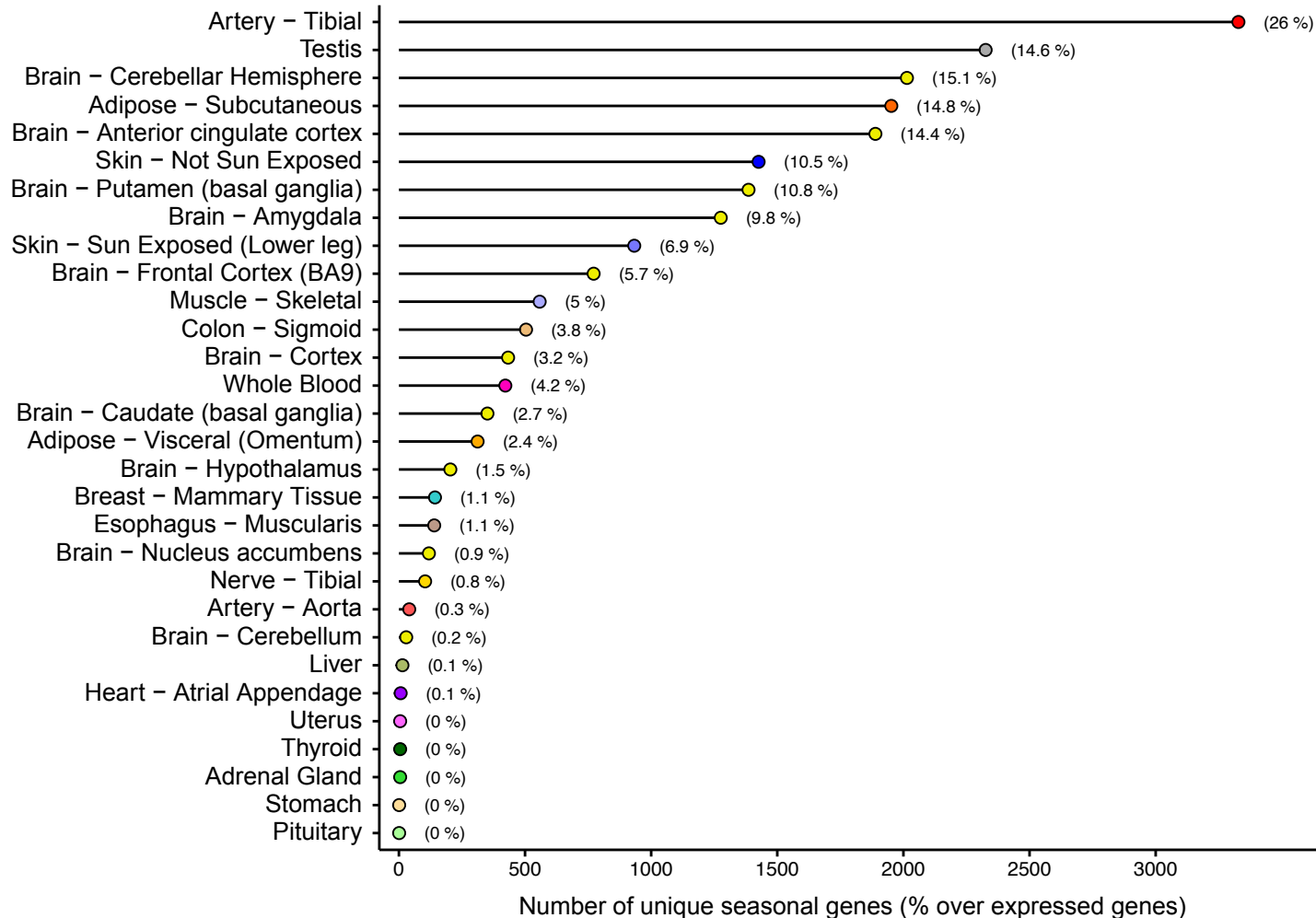

Supplement: S13 Fig — Number of unique genes found as seasonal (x-axis), i.e., genes differentially expressed in at least one season when compared to the others using FDR ≤ 0.1 as cutoff, per tissue (y-axis). The numbers in parentheses represent the percentage of unique seasonal genes in a given tissue over the number of expressed genes in that tissue. The data underlying this figure can be found in S1 Data. (PDF) [file pbio.3001986.s013.pdf]

Number of unique day-night genes

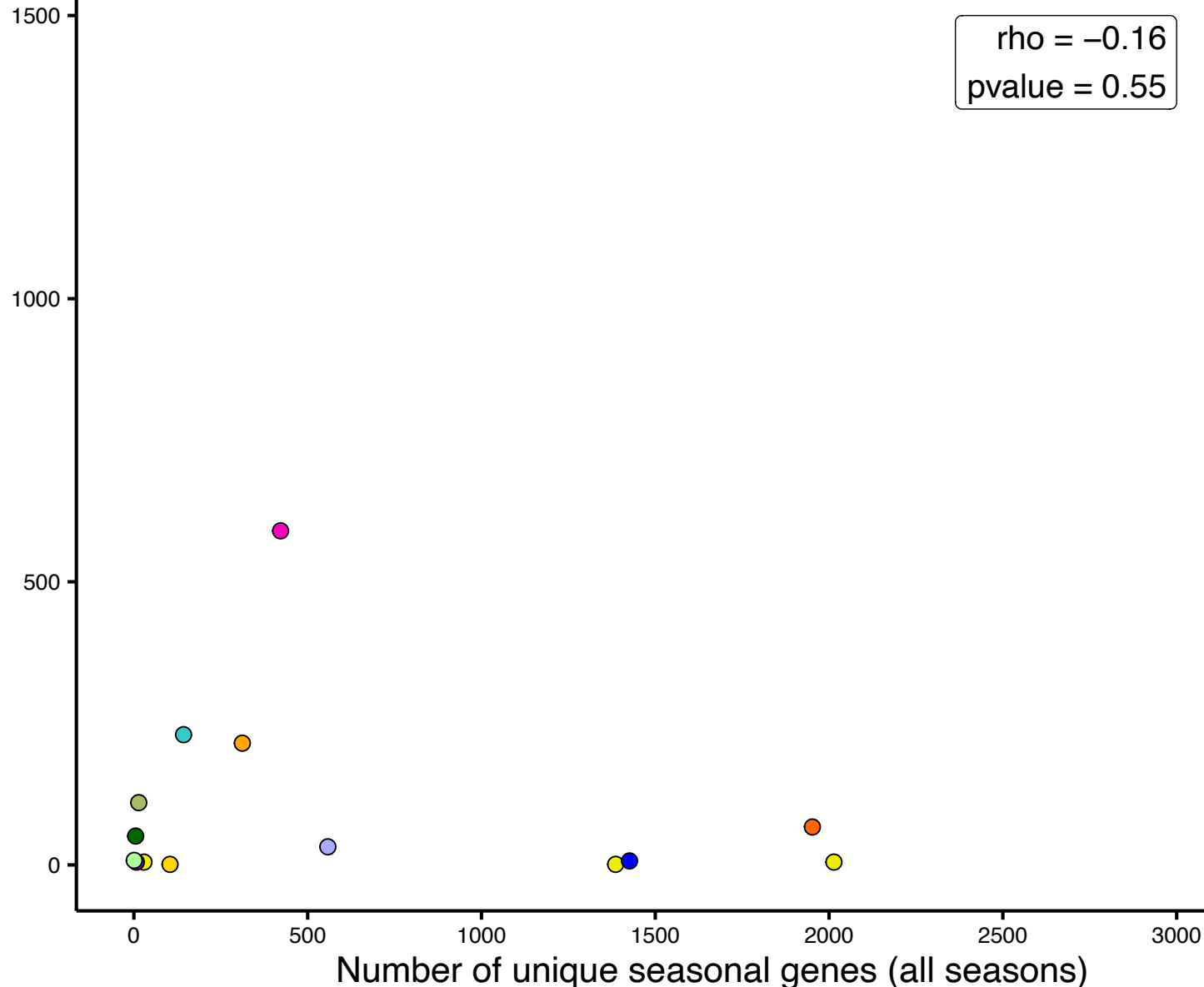

Supplement: S14 Fig — Statistics from a Spearman’s correlation is shown. Tissue colors correspond to the GTEx color panel. The data underlying this figure can be found in S1 Data. (PDF) [file pbio.3001986.s014.pdf]

**A*****RTF1* – Brain – Amygdala**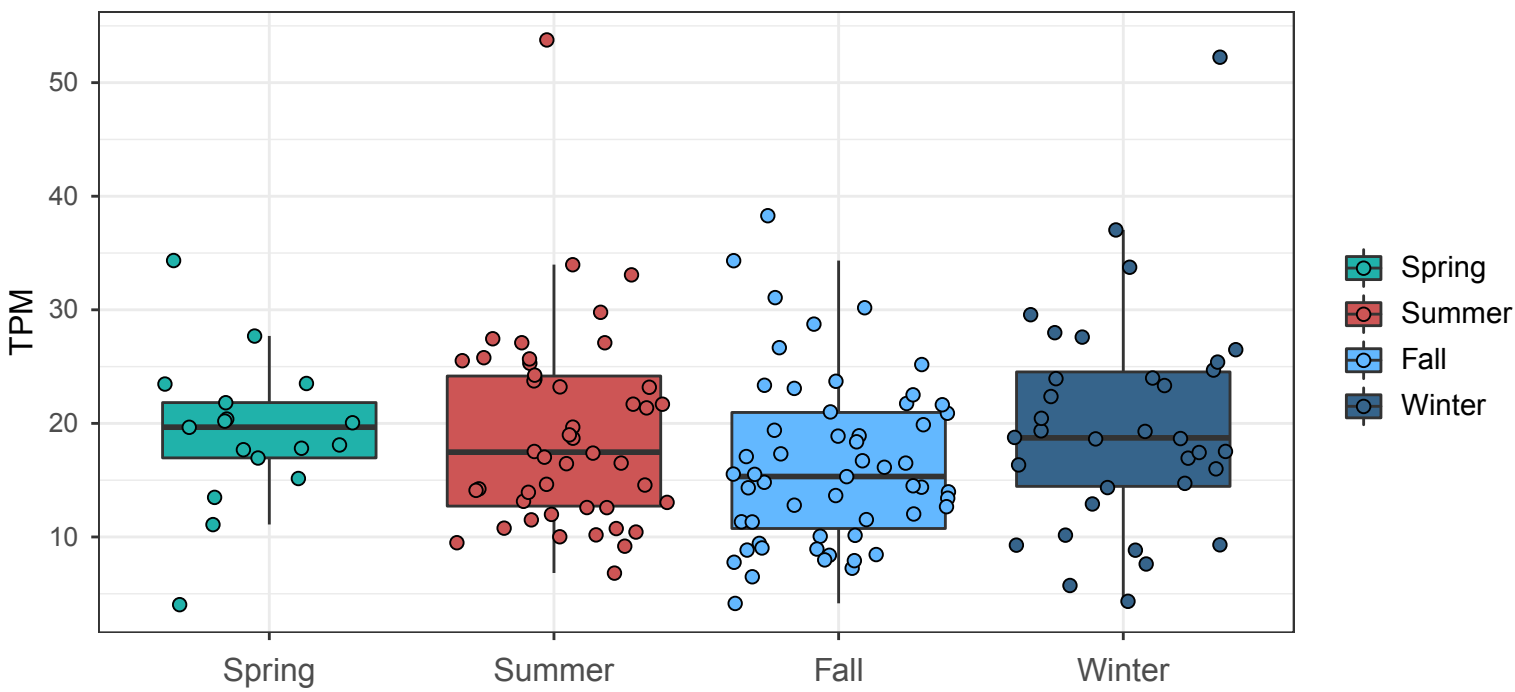**B*****C4A* – Adipose – Visceral (Omentum)**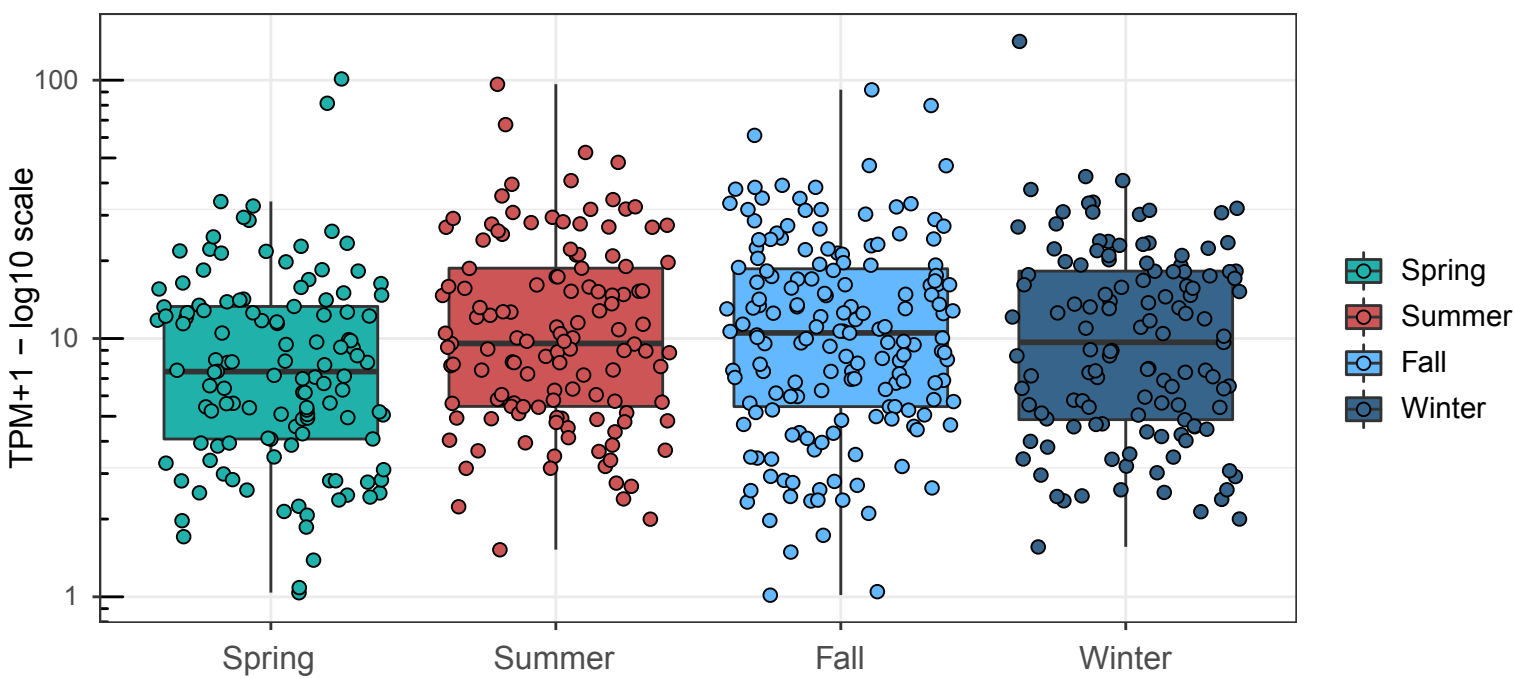**C*****KRT1* – Liver**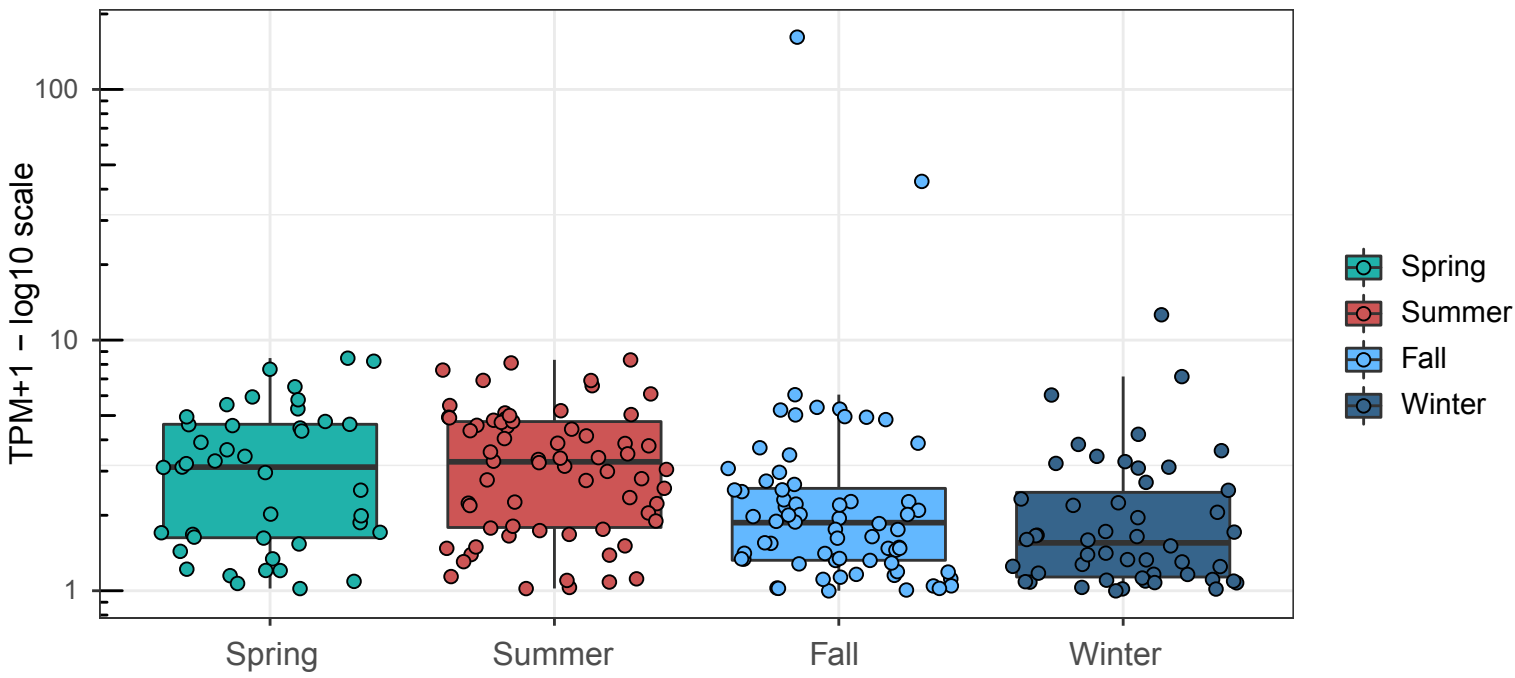

Supplement: S15 Fig — Boxplot of the TPM values on a log10 scale of three top seasonal genes in a tissue where they are differentially expressed: RTF1, underexpressed in fall (A), C4A underexpressed in spring (B), and KRT1 underexpressed in winter (C). The data underlying this figure can be found in S1 Data. (PDF) [file pbio.3001986.s015.pdf]

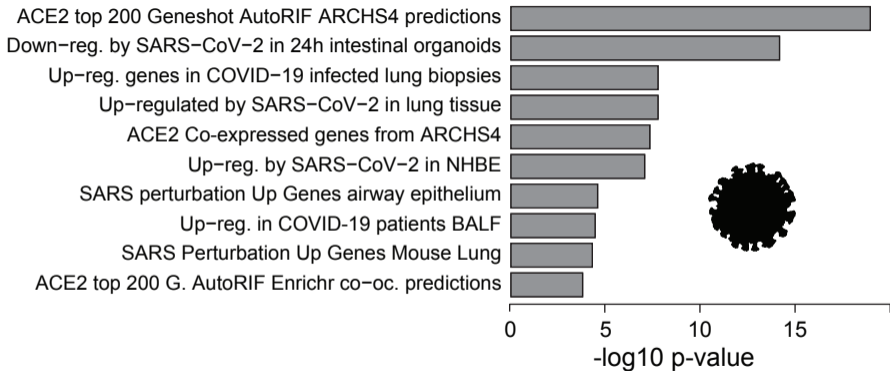

Supplement: S16 Fig — ACE2 Geneshot AutoRIF ARCHS4 predictions were obtained by Geneshot, combining genes previously published to be associated with ACE2, as well as genes predicted to be associated with ACE2 based on data integration from multiple sources, including coexpression matrices based on RNA-seq data and others. (PDF) [file pbio.3001986.s016.pdf]

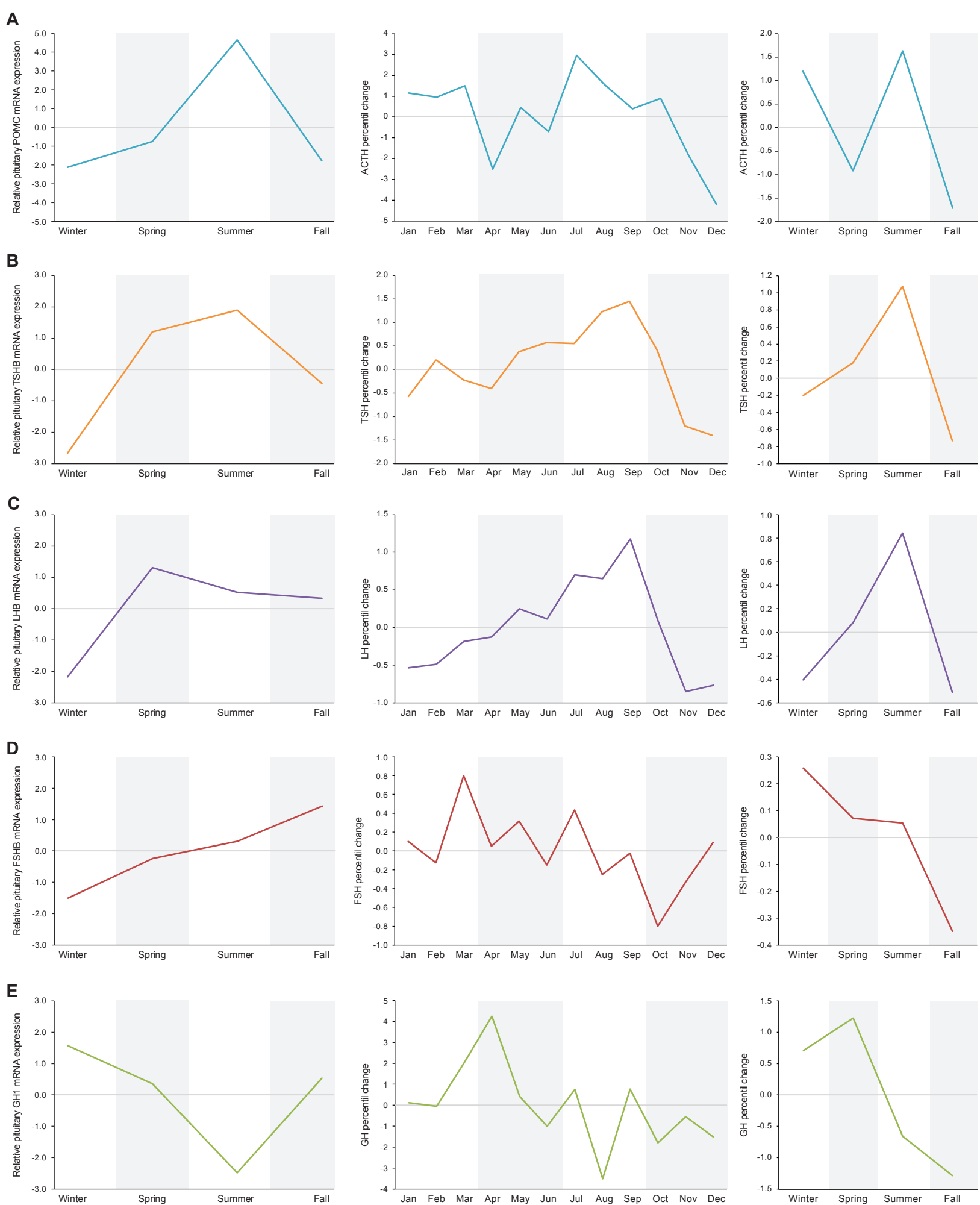

Supplement: S17 Fig — Left: Median mRNA expression levels for pituitary hormone genes in each season from GTEx. Centre: Average of male and female median values for hormones from Tendler and colleagues [43] obtained from Clalit medical records. Right: Average values group by season (Winter: Jan, Feb, Mar; Spring: Apr, May, Jun; Summer: Jul, Aug, Sep; Fall: Oct, Nov, Dec). Genes/hormones correspond to POMC/ACTH (A), TSHB/TSH (B), LHB/LH (C), FSHB/FSH (D), and GH1/GH (E). The data underlying this figure can be found in S1 Data. (PDF) [file pbio.3001986.s017.pdf]

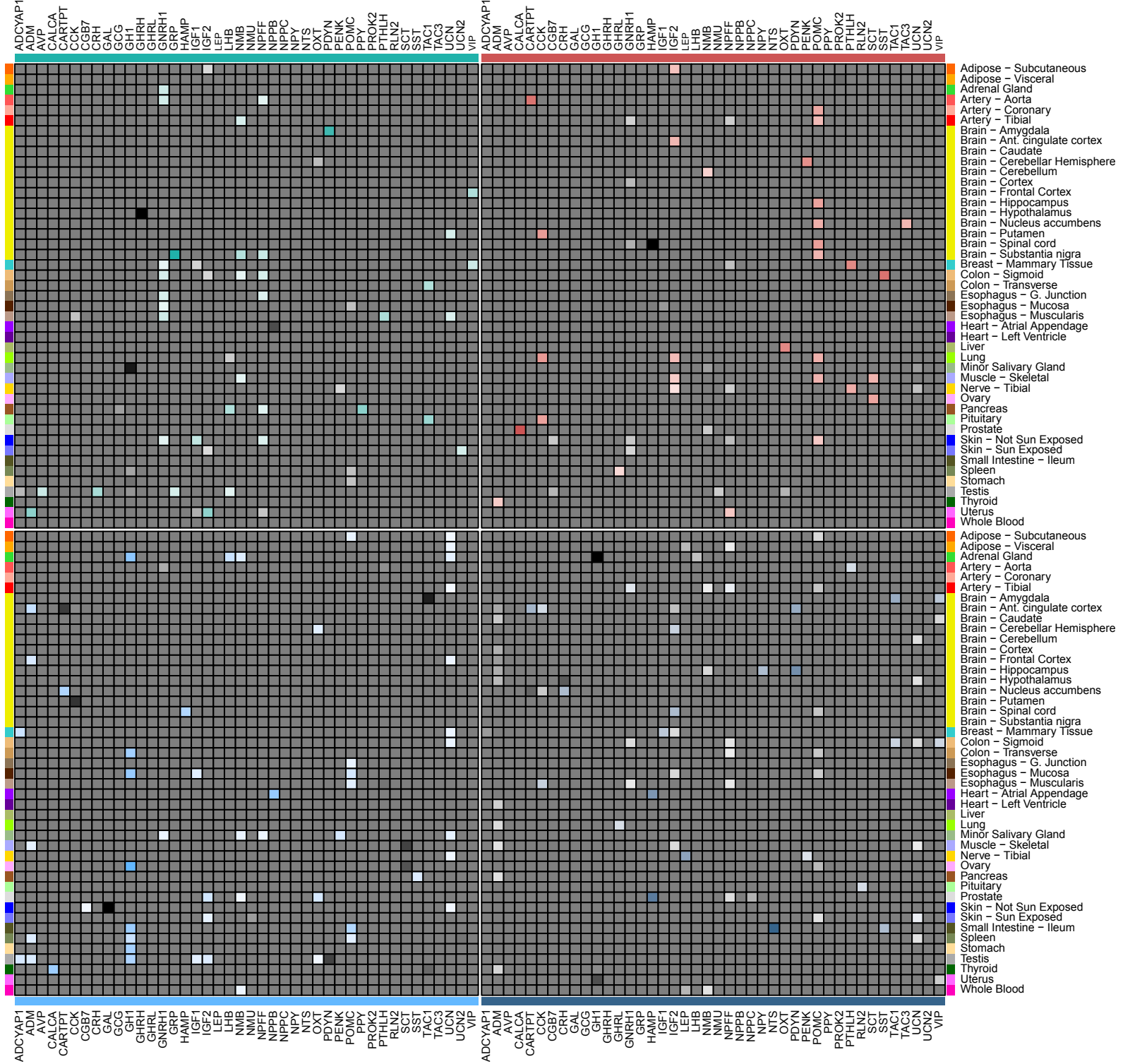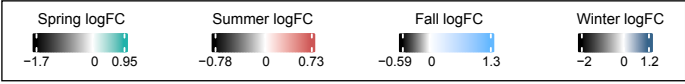

Supplement: S18 Fig — Seasonal log2 fold-change for the 42 hormone genes in the 45 tissues (all except vagina) with at least one hormonal gene showing seasonal expression. The data underlying this figure can be found in S1 Data. (PDF) [file pbio.3001986.s018.pdf]

A

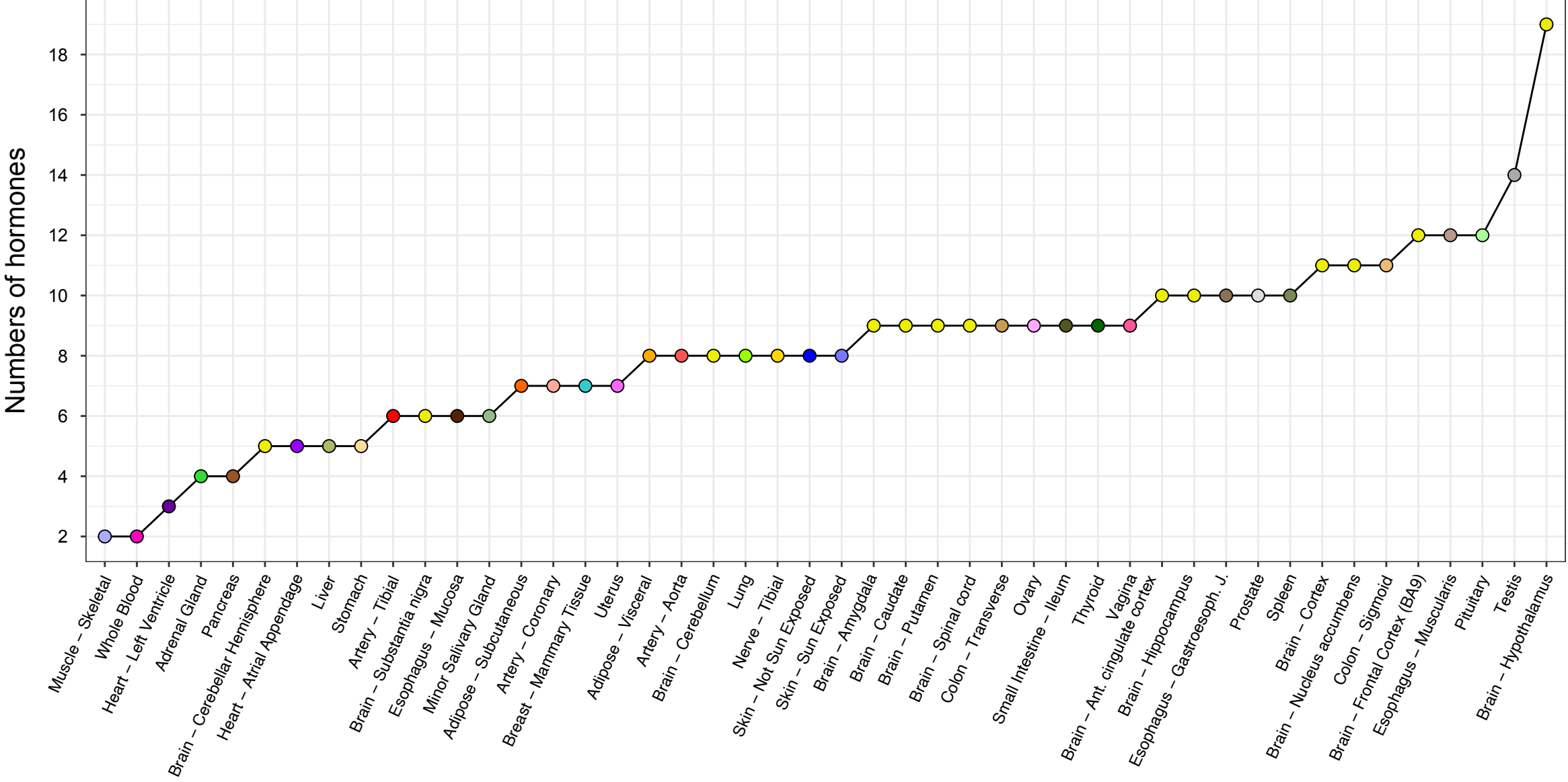

B

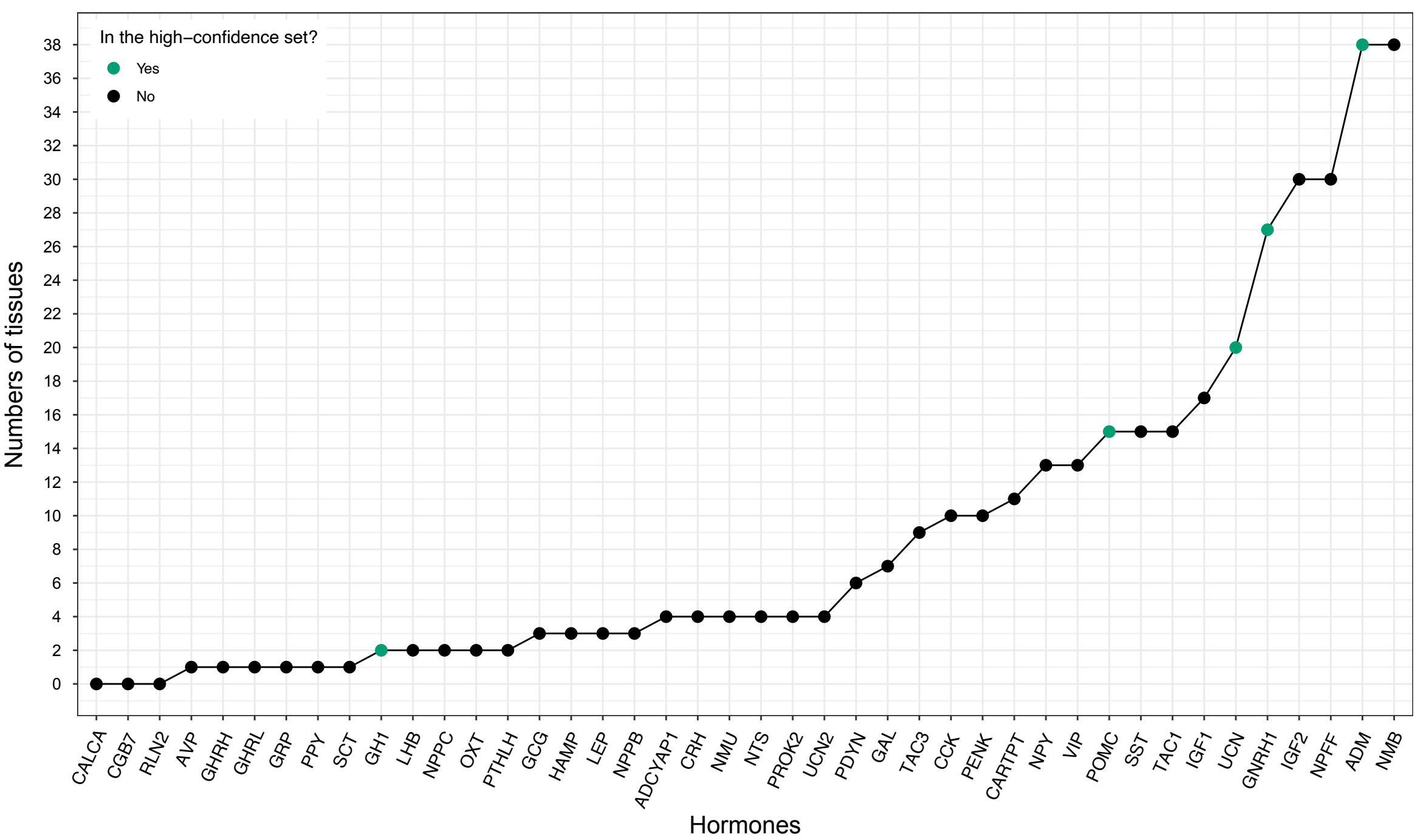

Supplement: S19 Fig — (A) For each tissue, the number of seasonal hormone genes with a median expression TPM ≥ 5. (B) For each seasonal hormone gene, the number of tissues for which the gene had a median expression TPM ≥ 5. The data underlying this figure can be found in S1 Data. (PDF) [file pbio.3001986.s019.pdf]

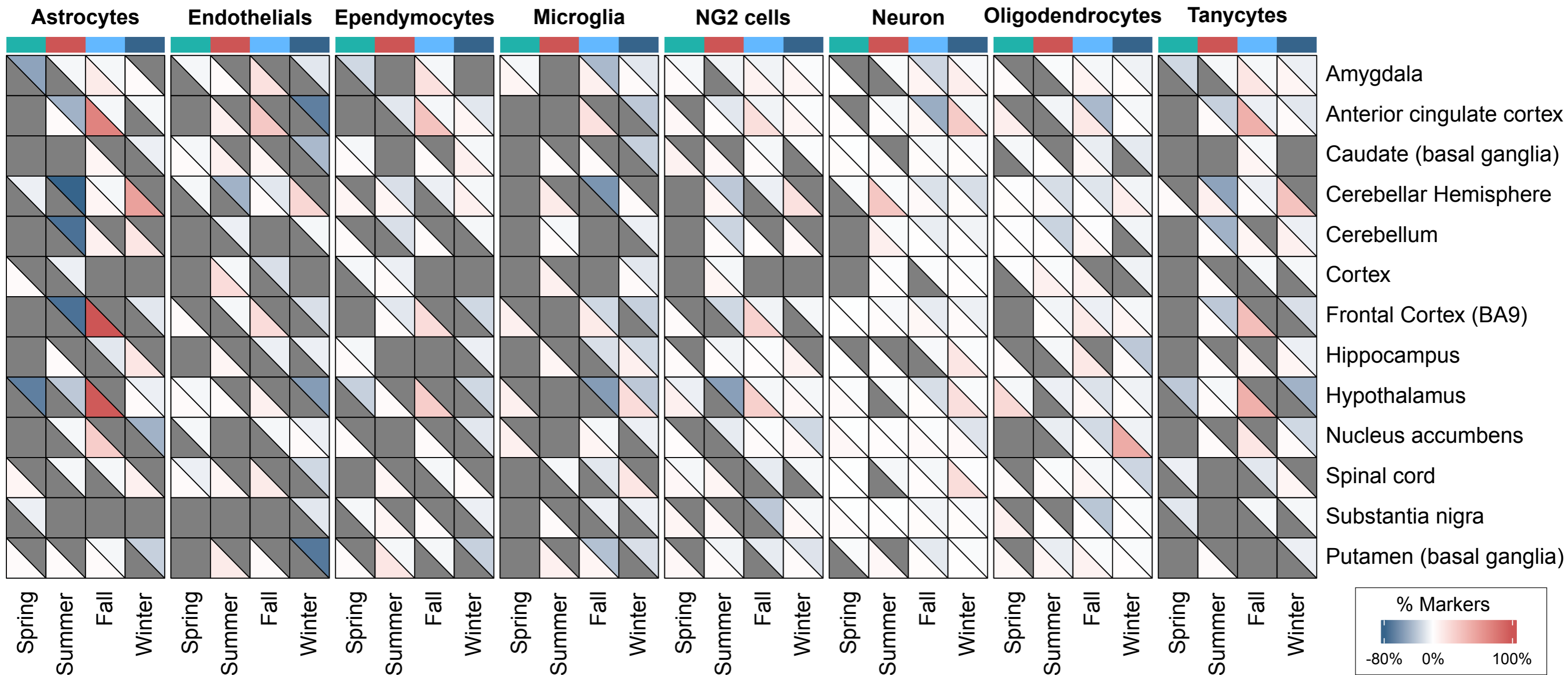

Supplement: S20 Fig — Percentage of the marker genes down-regulated (blue) or up-regulated (red) for major cell types identified by Wen and colleagues [60] in brain subregions (depicted in the brain scheme in Fig 6). Markers without significant effects were colored in grey. The data underlying this figure can be found in S1 Data. (PDF) [file pbio.3001986.s020.pdf]

**A**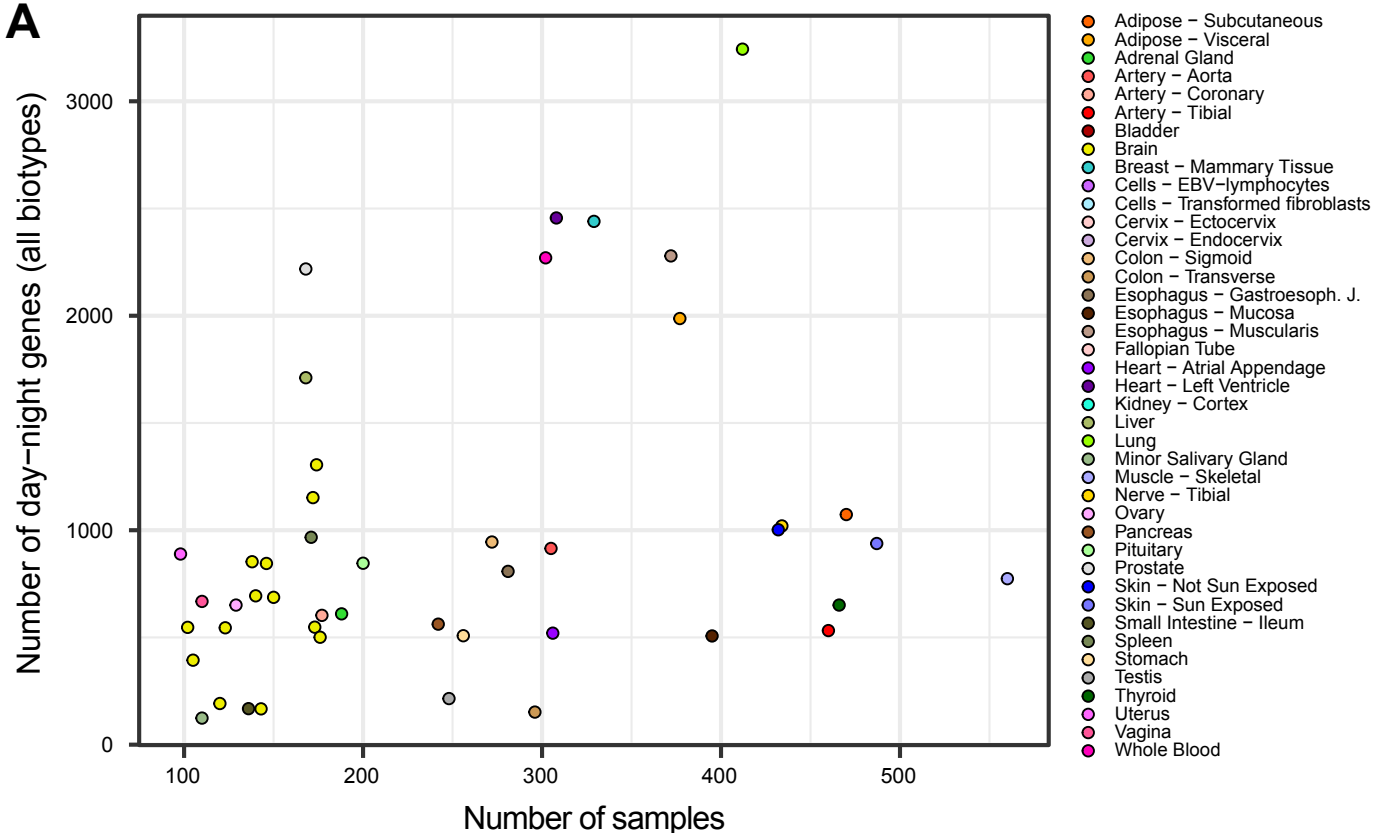**B**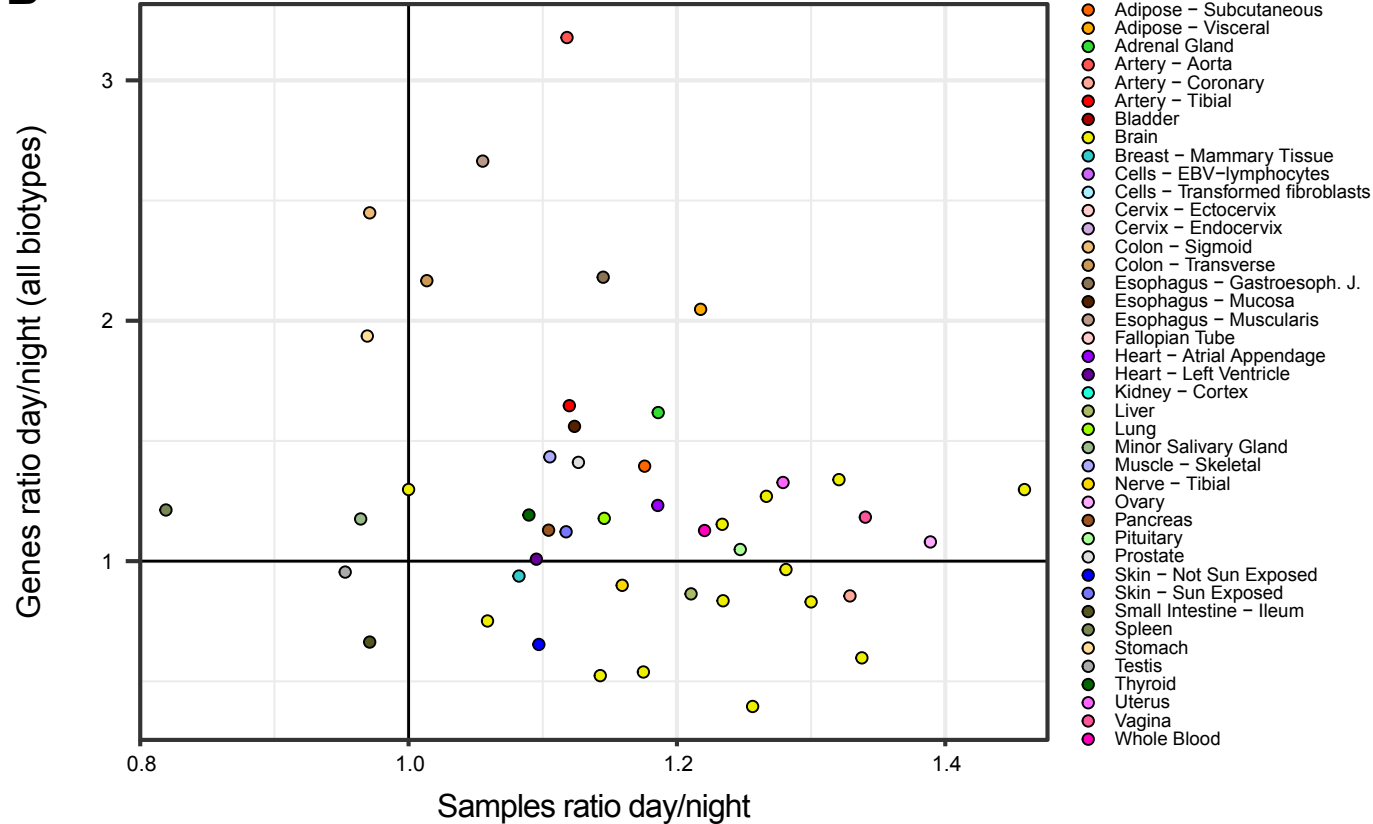

Supplement: S21 Fig — (A) Number of day-night genes per number of samples. (B) Ratio of the number of day over the number of night genes vs. the ratio of day over night samples. The data underlying this figure can be found in S1 Data. (PDF) [file pbio.3001986.s021.pdf]

**A**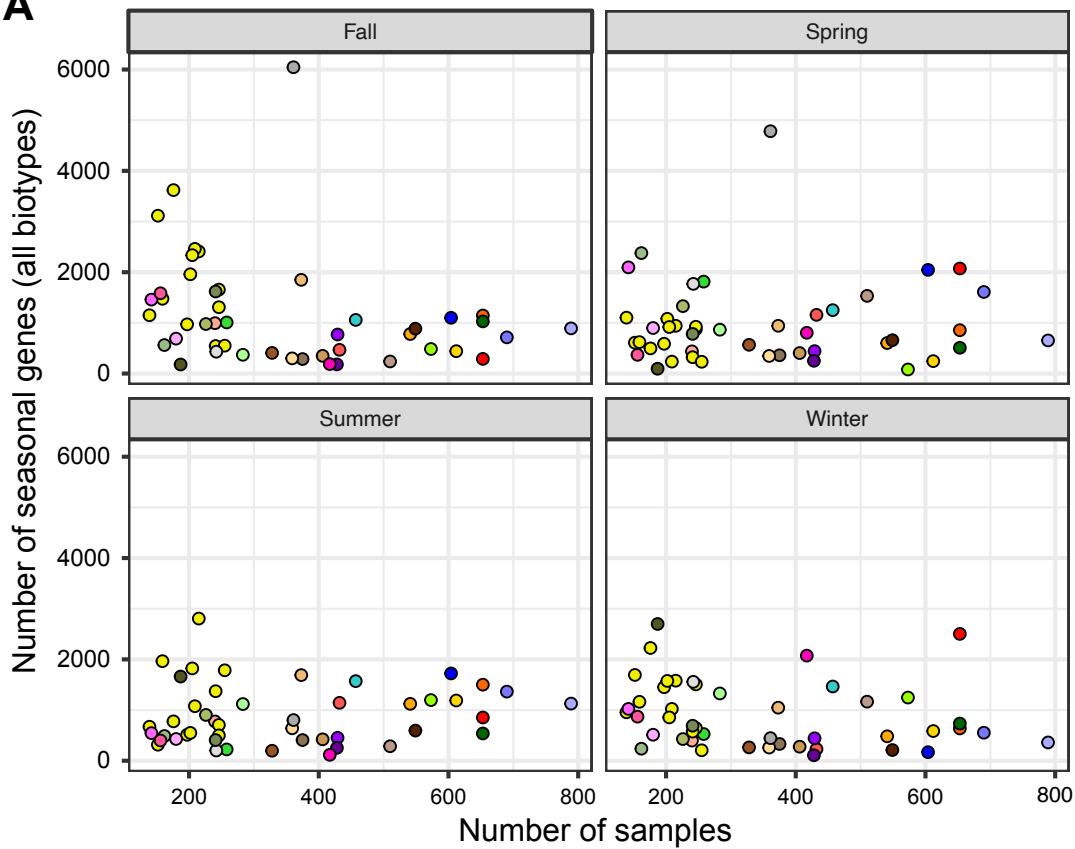**B**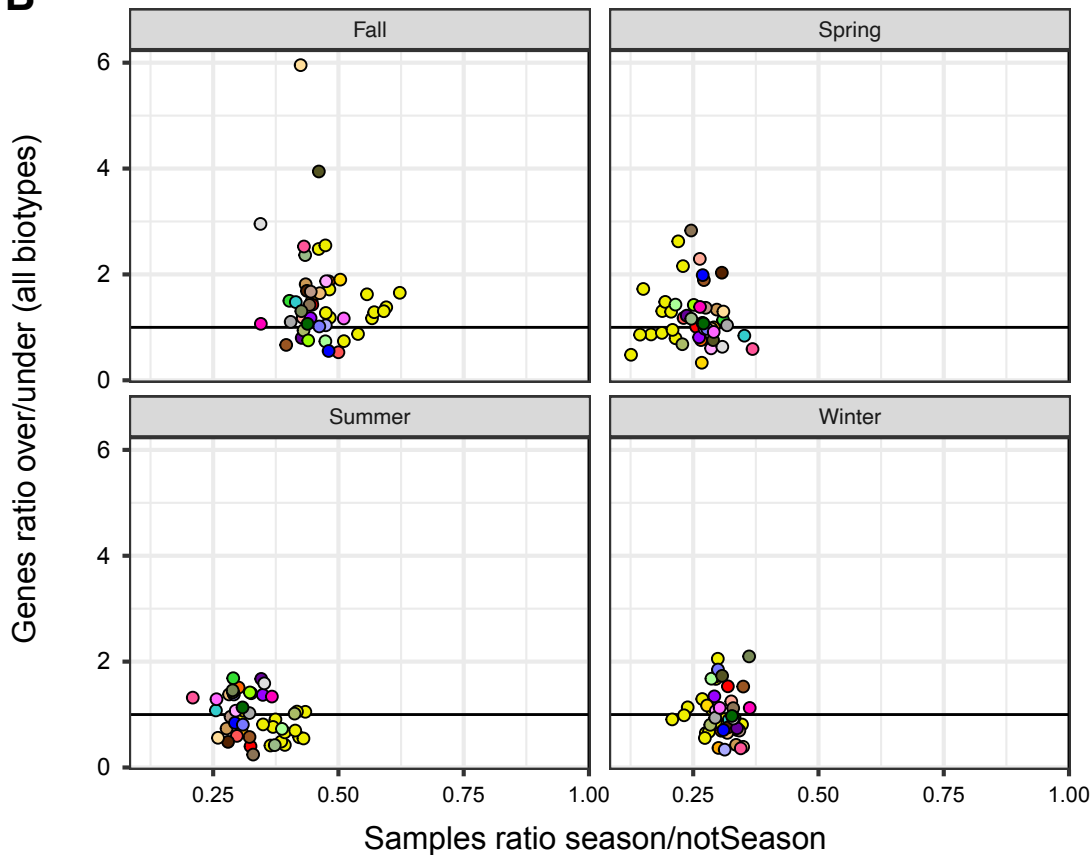

Supplement: S22 Fig — (A) Number of seasonal genes vs. number of seasonal samples per season. (B) Ratio of the number of genes found overexpressed over genes found underexpressed per season vs. the ratio of the samples from a specific season over all samples. The data underlying this figure can be found in S1 Data. (PDF) [file pbio.3001986.s022.pdf]
