# Supplementary material for: Day-night and seasonal variation of human gene expression across tissues
Source: PLoS Biol. 2023 Feb 6;21(2):e3001986. doi: 10.1371/journal.pbio.3001986 (PMC9934459; doi:10.1371/journal.pbio.3001986)
Supplement: S8 Fig — Number of sleep day-night genes (y-axis) vs. total day-night genes (x-axis) per tissue. The data underlying this figure can be found in S1 Data. (PDF) [file pbio.3001986.s008.pdf]

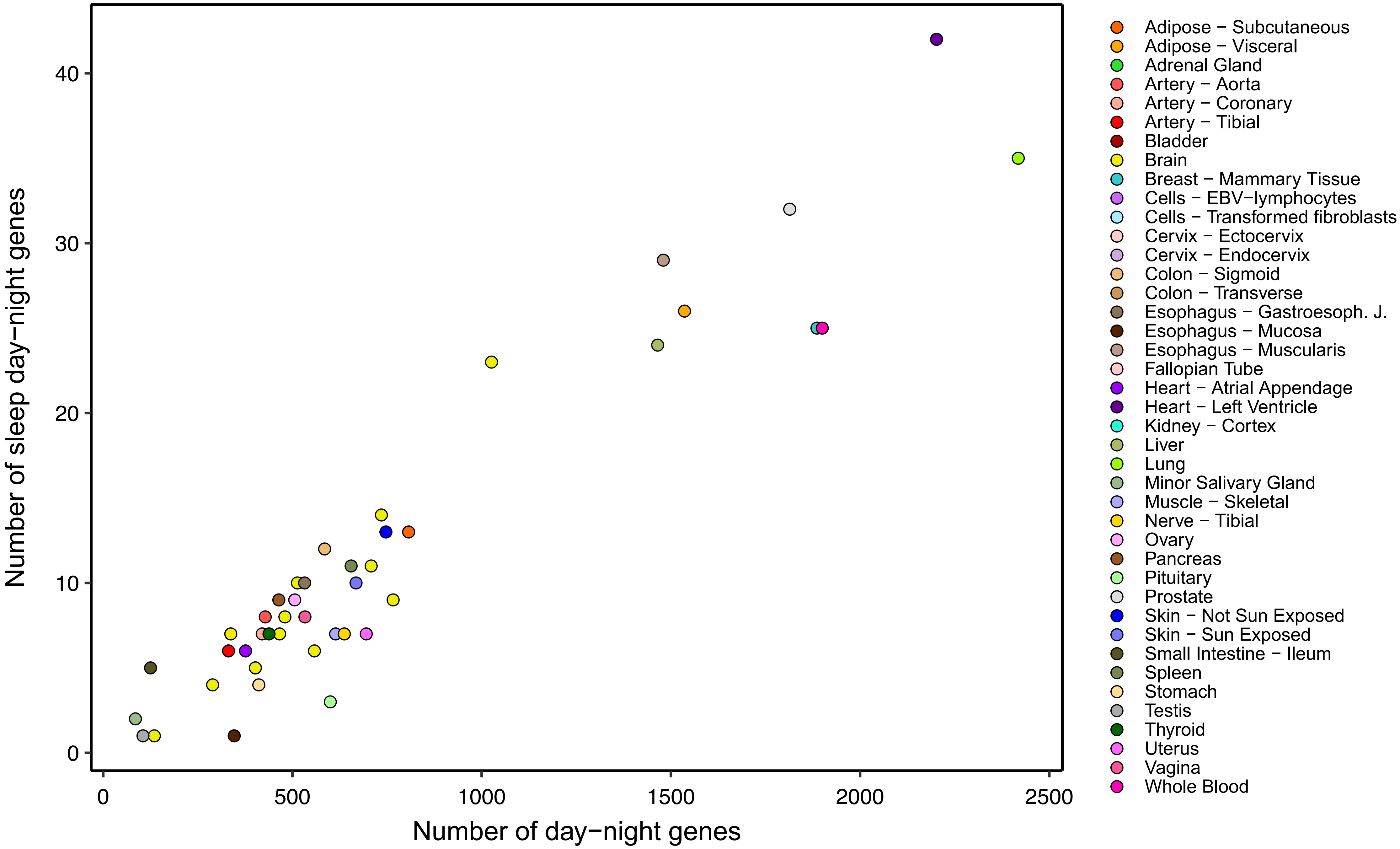

Number of sleep day−night genes
0
10
20
30
40
0
500
1000
1500
2000
2500
Number of day−night genes
Adipose − Subcutaneous
Adipose − Visceral
Adrenal Gland
Artery − Aorta
Artery − Coronary
Artery − Tibial
Bladder
Brain
Breast − Mammary Tissue
Cells − EBV−lymphocytes
Cells − Transformed fibroblasts
Cervix − Ectocervix
Cervix − Endocervix
Colon − Sigmoid
Colon − Transverse
Esophagus − Gastroesoph. J.
Esophagus − Mucosa
Esophagus − Muscularis
Fallopian Tube
Heart − Atrial Appendage
Heart − Left Ventricle
Kidney − Cortex
Liver
Lung
Minor Salivary Gland
Muscle − Skeletal
Nerve − Tibial
Ovary
Pancreas
Pituitary
Prostate
Skin − Not Sun Exposed
Skin − Sun Exposed
Small Intestine − Ileum
Spleen
Stomach
Testis
Thyroid
Uterus
Vagina
Whole Blood
