# Supplementary material for: Day-night and seasonal variation of human gene expression across tissues
Source: PLoS Biol. 2023 Feb 6;21(2):e3001986. doi: 10.1371/journal.pbio.3001986 (PMC9934459; doi:10.1371/journal.pbio.3001986)
Supplement: S9 Fig — Expression values (TPMs) for the four sleep-related genes in the day-night high-confidence gene set and annotated in the Circadian Gene DataBase: (A) PC in cerebellar hemisphere, (B) PITPNC1 in anterior cingulate cortex, (C) PDE4B in subcutaneous adipose, and (D) QSOX2 in pituitary at the time of death of the GTEx donors. The colors of the dots represent the classification of the individuals according to the time of death of the donor: during the day (yellow), during the night (blue), or in-between for twilight (grey). The samples classified as twilight have been discarded for the day-night analysis. The “circadian” curve was created using the geom_smooth function from ggplot2 in R with the “loess” method. The data underlying this figure can be found in S1 Data. (PDF) [file pbio.3001986.s009.pdf]

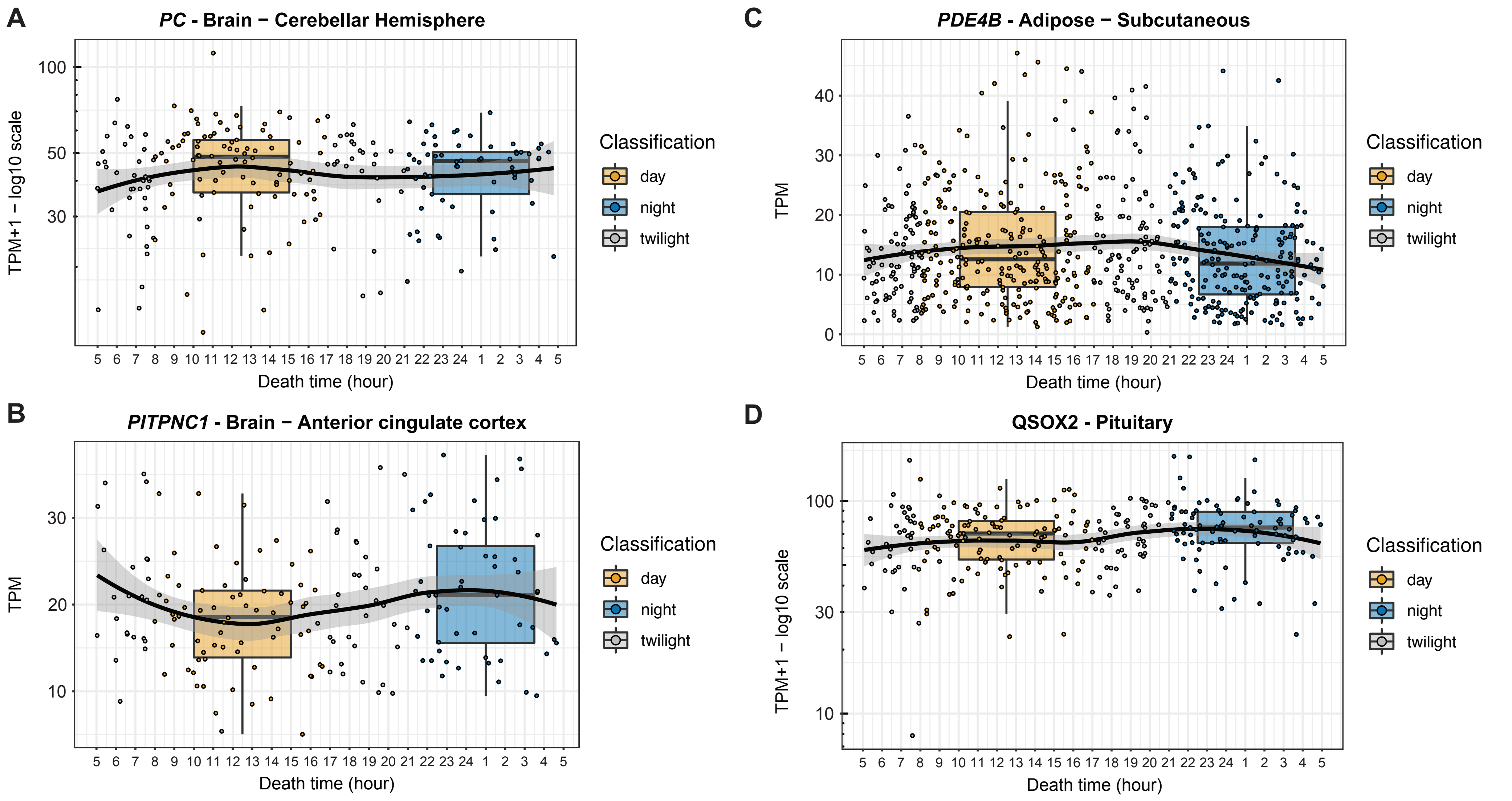
A
PC - Brain − Cerebellar Hemisphere
TPM+1 − log10 scale
100
50
30
Death time (hour)
5 6 7 8 9 10 11 12 13 14 15 16 17 18 19 20 21 22 23 24 1 2 3 4 5
Classification
day
night
twilight
C
PDE4B - Adipose − Subcutaneous
TPM
40
30
20
10
0
Death time (hour)
B
PITPNC1 - Brain − Anterior cingulate cortex
TPM
30
20
10
Death time (hour)
D
QSOX2 - Pituitary
TPM+1 − log10 scale
100
30
10
Death time (hour)
