# Supplementary material for: Day-night and seasonal variation of human gene expression across tissues
Source: PLoS Biol. 2023 Feb 6;21(2):e3001986. doi: 10.1371/journal.pbio.3001986 (PMC9934459; doi:10.1371/journal.pbio.3001986)
Supplement: Attachment — Submitted filename: PLOSBIO_3.pdf [file pbio.3001986.s048.pdf]

The manuscript entitled “Day-night and seasonal variation of human gene expression across tissues ” found the circadian rhythmic genes (day-night gene) and circannual rhythmic genes (seasonal gene) by using GTEx data with the time of the day and season of their death using a total of 932 donors. While the majority of day-night genes identified by this study are consistent with those found in previous studies, a previously unknown day-night gene, THRA, is also identified. Interestingly, this study also analyzed the tissue-dependent characteristics of the day-night genes. For instance, brain subregions and other tissues have an opposite circadian phase of NR1D2. Furthermore, this study investigates the difference in clock gene expressions between diurnal and nocturnal animals. This study also found 16,408 seasonal genes, which were still largely unknown, based on the statistical significance of the difference in gene expression with different seasons. However, the approach used in this study to detect day-night and seasonal genes has some limitations, which can be improved.

Major:

Page 3, Paragraph 3: In this study, the Lomb-Scargle (LS) method was used to detect rhythmic genes. However, LS is known to have poor performance in detecting rhythmic genes and low reproducibility (Wenwen Mei, 2021 doi: 10.1093/bib/bbaa135). Therefore, please use the other methods to make sure the analysis with LS is valid. For instance, BIO_CYCLE is more accurate than the LS method. Furthermore, it can be applied to data sets with uneven time intervals, which is important for this study.

Page 4, Paragraph 1: To obtain the day-night genes, only gene expressions in the day time range [08:00-17:00) and the night time range [21:00-05:00) were compared. The interval choice seems too subjective, and even the length of the two intervals is not the same. Furthermore, the approach seems inefficient because, i.e., the twilight data (222 individuals) was not used. Importantly, this leads to the biased selection of rhythmic genes. Specifically, if the genes’ rhythm has max at 19:00 or 7:00, the current approach cannot detect it as the day-night genes. Here is an alternative approach. Let’s set up two moving windows such as [i, i+9] and [i+11, i+19] for i=0,1,2,3,… and then identify genes having different expressions between two intervals for any choice of i. In this way, a larger number of day-night genes can be identified.

By comparing the number of rhythmic genes, the author evaluates the similarity between a baboon and human day-night genes (4p2p7l) or between seasonal genes or between seasonal genes and day-night genes (8p2p). However, comparing the number of genes is too simplistic to evaluate the similarity between the two groups. Instead, please use the Jaccard index; you can more accurately check the similarity between two groups of rhythmic genes.

Page 8, Paragraph 1: The number/fraction of seasonal genes is much higher than those of day-night genes. However, this result seems biased due to the different analysis methods used to detect seasonal and day-night genes. For the seasonal genes, if the expression of a gene of the four windows across the season is distinguished, it is considered a seasonal gene. On the other hand, for day-night genes, two windows are used. Of course, the chance that one of four is distinguished is higher than one of two. Please perform a consistent analysis. Specifically, the moving window approach with the two windows, suggested above, can be used for the seasonal gene detection to test whether the still the number of seasonal genes are higher than the number of day-night genes and there is no correlation between them across tissues.

Page 12 Discussion: In the previous study, the time of death was inferred from the expression of known circadian genes. On the other hand, in this study, the actual death time is used. Please investigate how such times are different for each death case (highly correlated?). If they are not highly correlated, please justify the choice of the time.

Minor:

Page 7 Paragraph 2: 254 genes known to be related to insomnia are considered. Are there any genes related to circadian rhythms sleep disorders?

Page 11 Paragraph 2: The authors analyzed seasonal differences in cell-type-specific markers in the brain. In a recent study (Shao'ang Wen, Nat. Neuroscience, 2020), key marker genes of sub-populations of SCN have been reported. Please discuss whether those marker genes are included in the identified day-night genes/seasonal genes in the brain.

It is well known that the photo-period varies depending on the season, affecting the core clock genes of the Suprachiasmatic nucleus (SCN), the "master clock" of mammals. (Evans et al. BMC Biology, 2015, Gerald Lincoln, PNAS, 2002, Z. BENDOVÁ, Physiol. Res, 2006). It would be interesting to check whether expression levels of day-night genes change depending on the season. In particular, please check whether the expression of the core clock genes shows any seasonality.

Page 20: Why are the numbers of the marker genes for microglia (9) and neuron (11) set differently from the numbers of other marker genes (10)?

Discussion: When a drug targets molecules whose level changes in a circadian manner, the efficacy of the drug can be changed depending on the dosing time of day (i.e., chronotherapy) (https://doi.org/10.1038/s41574-018-0142-x , https://doi.org/10.15252/msb.20198838). Similarly, when a drug targets seasonal genes, we can expect seasonality in drug efficacy. It would be great to discuss whether the identified day-night genes and seasonal genes in this study are targets of drugs, as done in Zhang et al. PNAS (ref 1 of the manuscript).
