# Supplementary material for: Day-night and seasonal variation of human gene expression across tissues
Source: PLoS Biol. 2023 Feb 6;21(2):e3001986. doi: 10.1371/journal.pbio.3001986 (PMC9934459; doi:10.1371/journal.pbio.3001986)
Supplement: Attachment — Submitted filename: Point-by-point_response_reviewers-R1.pdf [file pbio.3001986.s049.pdf]

**Reviewer #1**

The authors exploit the valuable resource of GTEx expression profiles from human tissues with respect to day-night differences and seasonal variations. There are related studies such as Ruben 2019 and Talamanca (BioRxiv) but Wucher et al. apply specific bioinformatics methods to mine the exciting database. Instead of extractions of circadian phases and amplitudes as in the other studies, they analyze expression levels between day and night and across seasons with established statistical tools. They find novel hormonal and immunological regulations and seasonal variations of brain regions.

**Response**: We thank the Reviewer for their positive comments about our manuscript and constructive suggestions.

**1.** The analysis is based on the time of death. Is there information available regarding the time span between death and RNA-extraction? Other groups (Ruben, Talamanca) exploit the core clock genes to define an internal time frame via CYCLOPS or Per3 expression. Is it possible to study phase shifts between time of death and these phase markes? Even though this might be an interesting topic for the discussion the analysis in the manuscript uses coarse-grained definitions of day and night and seasons and thus I do not expect major effects on the precise extraction of intrinsic time.

**Response**: Concerning the time span between death and RNA-extraction, GTEx metadata contains the tissue post-mortem interval, which is the time between death and when the RNA of the tissues has been extracted and frozen. This time interval is included in our statistical model to define day-night and seasonal genes. Moreover, in Pedro *et al.* (2018), we showed that the post-mortem interval has an effect on fewer than 200 genes for most tissues.

We agree with the Reviewer that, in principle, it would have made sense to include in the manuscript a comparison between our results and those obtained using CYCLOPS by Ruben *et al.*. Since this study provides a compendium of genes with circadian expression inferred from the GTEx data, one would expect a high degree of overlap. However, the authors of CYCLOPS did not use the donor's time of death reported in the metadata, but they inferred it from the expression values of known circadian genes, as explained by the Reviewer. They assign to each sample (tissue and individual) a phase from 0 to 2π, and the phases were adjusted so that the peak of the gene *BMAL1* (*ARNTL*) is at phase 0 (which corresponds to 9PM). While this is, in principle, a nice approach, we observed a series of problems. First, in some cases, the inferred phase is actually different for different tissues from the same individual. Second, and more importantly, most samples were inferred by CYCLOPS to be at phase 0 = 2π, while the GTEx metadata shows a relatively even distribution of times of death (Figure R1 below). Therefore, not surprisingly, we found a poor correlation between the CYCLOPS inferred time of the samples and the GTEx reported time of death.

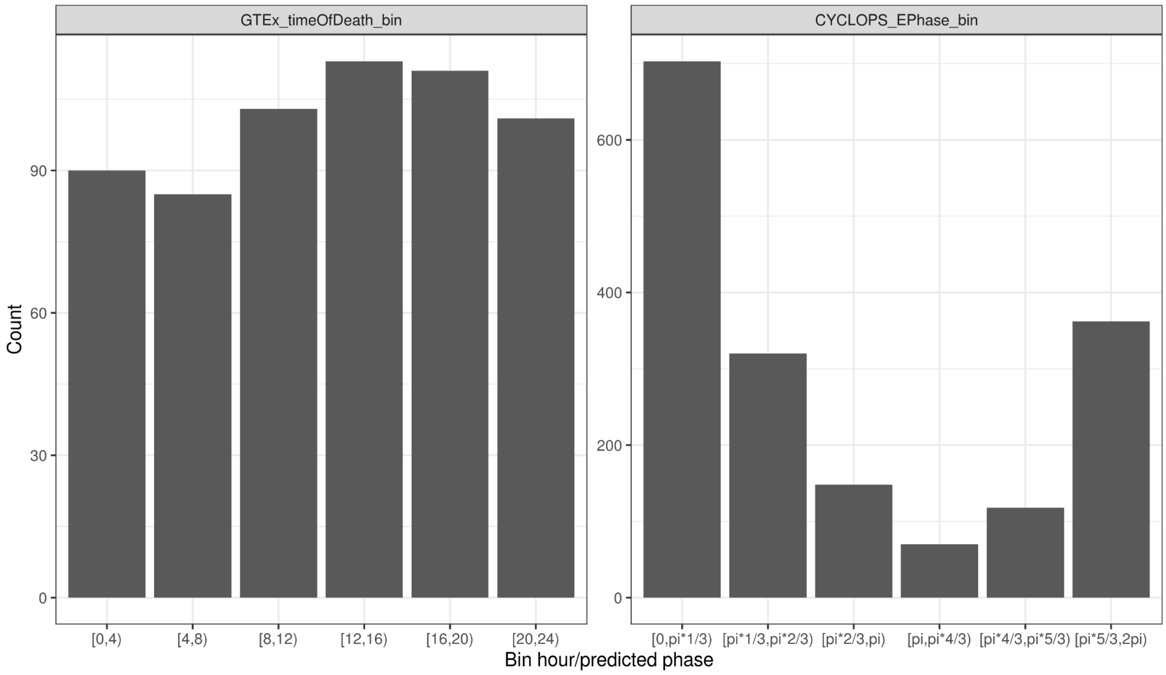


***Figure R1:** Distribution of GTEx samples based on the reported time of death (left) and the estimated time by CYCLOPS (right).*

Since in our analysis we are using the actual time of death reported by GTEx, our time frame is essentially unrelated to that of CYCLOPS and we do not think that a direct comparison between the CYCLOPS circadian genes and our day-night genes would be fair for the CYCLOPS study, which did not have access to the information regarding the time of death.

**2.** The authors apply the most appropriate and up-to-date bioinformatics methods: MetaCycle, Lomb-Scargle, voom/limma, permutations of data, multiple testing corrections. I found it helpful that different levels of sensitivity have been used.

Many results are consistent with previous findings of Ruben et al. Interestingly, NR1D2 has different behavior in brain and other tissues. This is relevant since NR1D2 is used often in body-time studies (see e.g. Witenbrink 2018). Another interesting finding is the association with insomnia genes.

To me, the seasonal overexpression studies are really novel. Interestingly, they seem weakly related to diurnal rhythms (see Fig. 4B). As expected there are interesting relations to the yearly rhythms of specific hormones.

It is intriguing to see differentials of Clock and Npas2 in rodents and humans. It is known that light and feeding are out-of-phase but melatonin is peaking in the dark in both, in nocturnal and diurnal animals. Is there any link between melatonin and Clock or Npas2 known?

**Response**: We are glad the Reviewer appreciates the contribution of our work. Regarding Clock/Npas2 and melatonin, in Haque *et al.* 2010 (PMID: 20345751), the authors showed in the chicken cone photoreceptors that *Aanat*, the main regulator of the circadian synthesis of melatonin, is regulated by *Clock* and *Npas2*. Specifically, they inhibited the expression of *Clock* or *Npas2* and they observed a reduction of *Aanat* expression. Therefore, this suggests that these regulatory interactions may not be conserved between human and mouse. We have added a small discussion on this point in the Discussion.

**Reviewer #2**

Although I think the authors has done important research and I have no doubt of the validity of the results, which do deserve publishing, in my opinion the results are too vague for PLOS Biology and would be more appropriate for PLOS1.

**1.** When trying to summarise in my head what are the most important conclusions of this research, the best I could do was the following:
1. There is a day/night and seasonal variation of gene expression, but different genes are expressed (variably on these factors) in different tissues.
2. The genes that are variably expressed with high confidence between day and night include known circadian genes, but there are others previously unknown; some of them, including the ones that are variably expressed in many tissues are hard to explain (such as ribosomal protein RPS26).
3. There is seasonal variation in hormonal and brain genes.

Have I missed something important? If not, I think that the authors will agree that this is a bit thin for PLOS Biology.

**Response:** We thank the Reviewer for their positive comments on the validity of our results.

Regarding the specific criticism, we would like to emphasize that, despite the various unavoidable limitations in the experimental design and available metadata, this is, and will be for many years, the only dataset available to perform population-wide multi-tissue transcriptomic studies of human phenotypes. Therefore, our work is, to our knowledge, the first human multi-tissue circannual transcriptomics study, and one of the first and most complete multi-tissue studies on human day-night variation. Also, it is the first one in which a direct comparison between the two types of variation has been performed. The resource generated provides information on genes that change expression during the day-night and/or seasonal cycles in different tissues, and conversely, on the tissues that are most affected by these cycles. In addition to its value as a resource, our work reports the following main findings:

1. Day-night and seasonal variation are largely uncoupled. Brain and gonadal tissues exhibited the highest seasonality, whereas those in the thoracic cavity showed stronger day-night regulation. Thus, not only genes, but also tissues, are differently affected by day-night and seasonal variation.
2. In addition to core clock genes, we found many other genes that show day-night variation, which were previously unknown. Therefore, this study opens new avenues for circadian genetic research.
3. Up- or down- regulation of gene expression during the day-night cycle is highly consistent across tissues, with remarkable exceptions, such as *NR1D2*, for which brain regions show the opposite phase than non-brain tissues (as highlighted by the other two Reviewers).
4. Tissue-dependent circadian variation of gene expression is conserved in mammals, but adapted to the preferential diurnal/nocturnal habits of the animals.
5. Seasonal variation of expression is enriched among genes associated with immune response, consistent with the seasonality of viral infections.
6. Seasonal variation of gene expression unveiled potential cytoarchitectural changes in brain subregions.
7. We provide examples of a number of human genes that change expression seasonally and discuss potentially relevant biological implications.
8. In this revised version of the manuscript, we also provide a list of day-night and seasonal genes that are drug targets (as suggested by Reviewer 3), with potential posological applications.

**2.** But moreover, I think there is one flaw that needs to be addressed before this can be considered further. As far as I was able to tell, no attempt has been made to separate the direct circadian variation from indirect effects of human day/night activities. For instance, one of the conclusions is that many genes have day/night variability in heart, lung and digestive tract. But humans are typically more active during the day, with higher heart rates and also more exercised lung. The eating patterns too are different, which may be expected to affect digestive tract. I do not know if heart gene expression is known to depend on the activity level, but I would be somewhat surprised if it was not. In any case, I think the authors need to look into these possible secondary effects. (At least in one instance they have done this for seasonal variation - there are more infections during winter, which affects the immune system). Possibly there is no sufficient metadata in GTEx to do this in-depth, but at least this has to be discussed. Perhaps, some of the genes they found can be explained by such secondary effects?

**Response:** We thank the Reviewer for this insightful and important comment. We would like to clarify that we do not claim that the observed day-night variation is all due to inner circadian regulation. Therefore, it is very possible that some of these differences in specific tissues are, as the Reviewer suggests, indeed due to other factors (as in the case of viral infections in winter or sunlight for exposed skin). This was already acknowledged in the original submission. For instance, we wrote: "Tissues with larger day-night than seasonal variation included various tissues from the thoracic cavity (e.g., lung and the heart's left ventricle), which may reflect changes in heart rate and breathing patterns between day and night". We have now made this point more explicit in the Discussion.

Unfortunately, however, there is no metadata in GTEx to directly tackle these factors. Nevertheless, we believe that this does not reduce the interest and impact of our findings given the explicit focus on day-night variation and the novelty of this approach. It should also be kept in mind that this focus makes the results more comparable to those obtained for seasonal variation.

**3.** On a related matter, perhaps the authors could also discuss some of the genes that are difficult to explain, such as RPS26. Can this be an artifact?

**Response:** While we understand that the significance of *RPS26* as a putative day-night gene can be difficult to interpret at this point and will require experimental testing, we in fact provided the high-confidence gene sets to minimize artefacts associated with specific tissues/samples. We ask the Reviewer to please bear in mind that our high-confidence gene set is composed of genes that are day-night across multiple tissues in which they show a strong and consistent behavior. In this specific case, *RPS26* shows a significant day-night variation in 16 tissues, and it is more highly expressed during the day in all 16/16 tissues. Therefore, we believe that the probability that this is a statistical artefact is minimal. One overarching goal by defining the high-confidence gene sets was to provide the scientific community with lists of interesting and reliable candidates for further studies on day-night and seasonal transcriptomic variation.

**Reviewer #3**

The manuscript entitled "Day-night and seasonal variation of human gene expression across tissues " found the circadian rhythmic genes (day-night gene) and circannual rhythmic genes (seasonal gene) by using GTEx data with the time of the day and season of their death using a total of 932 donors. While the majority of day-night genes identified by this study are consistent with those found in previous studies, a previously unknown day-night gene, THRA, is also identified. Interestingly, this study also analyzed the tissue-dependent characteristics of the day-night genes. For instance, brain subregions and other tissues have an opposite circadian phase of NR1D2. Furthermore, this study investigates the difference in clock gene expressions between diurnal and nocturnal animals. This study also found 16,408 seasonal genes, which were still largely unknown, based on the statistical significance of the difference in gene expression with different seasons. However, the approach used in this study to detect day-night and seasonal genes has some limitations, which can be improved.

**Response:** We thank the Reviewer for their comments and constructive feedback.

**Major**:

**1.** Page 3, Paragraph 3: In this study, the Lomb-Scargle (LS) method was used to detect rhythmic genes. However, LS is known to have poor performance in detecting rhythmic genes and low reproducibility (Wenwen Mei, 2021 doi: 10.1093/bib/bbaa135). Therefore, please use the other methods to make sure the analysis with LS is valid. For instance, BIO_CYCLE is more accurate than the LS method. Furthermore, it can be applied to data sets with uneven time intervals, which is important for this study.

**Response**: We thank the Reviewer for their expert comment on the Lomb-Scargle method as well as the suggestion to apply the BIO_CYCLE method. We applied BIO_CYCLE to our dataset using all samples per tissue. The numbers of significant genes per tissue provided by BIO_CYCLE can be seen in Figure R2 below. However, we can observe that the number of significant genes is very strongly driven by the number of samples available per tissue, unlike in our analysis (see Supplemental Fig. S21A). In addition, we were unable to find a way to include any variables/covariates to the BIO_CYCLE analysis (sex, age, body mass index, postmortem interval, and season of death), as we did for our approach. For these reasons, we believe that BIO_CYCLE is not a suitable method for our data, and we prefer not to include these results in our manuscript.

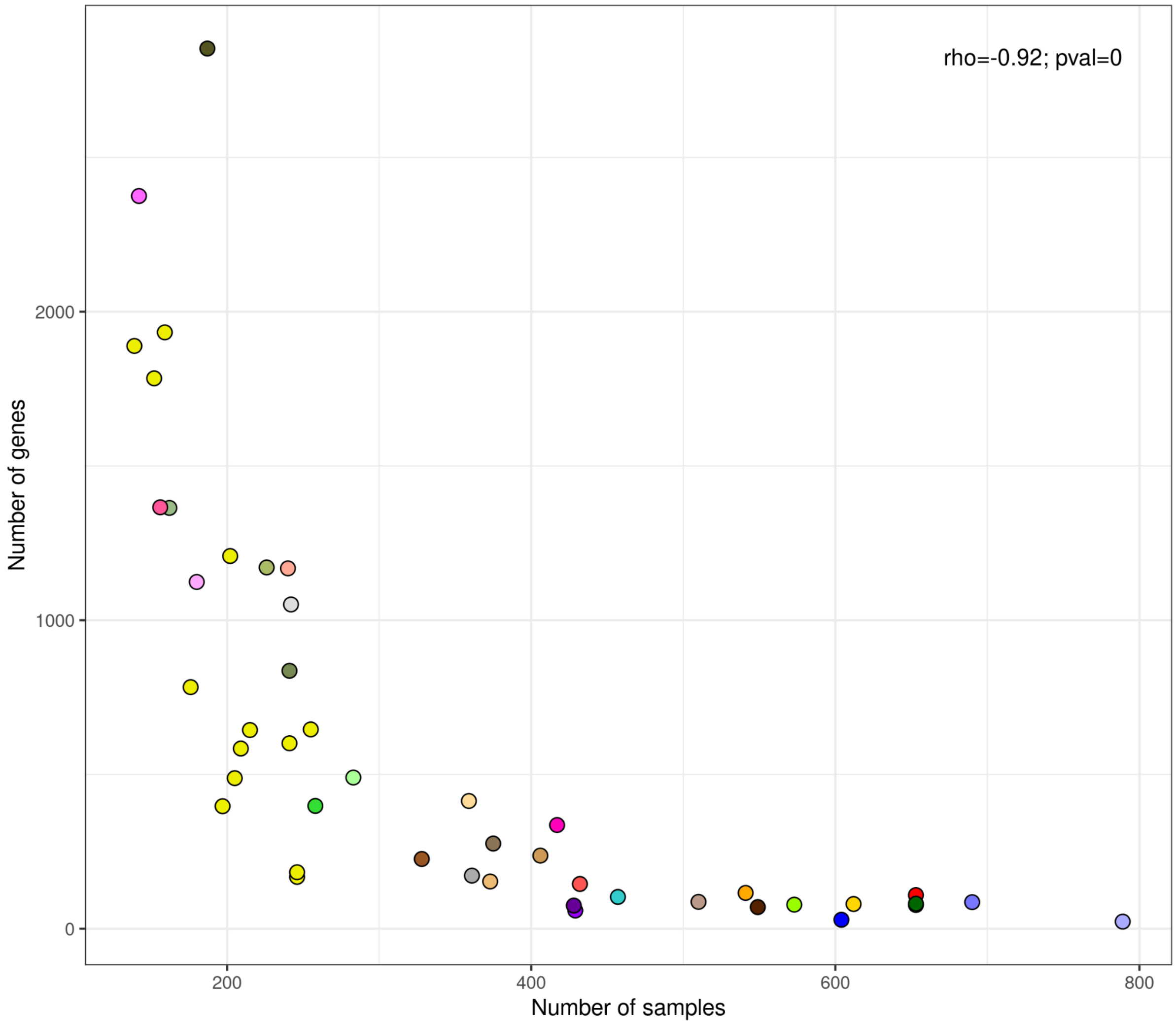


***Figure R2***: *Number of genes detected by BIO_CYCLE (y-axis) per number of samples by tissues. The Spearman rho is added on the top-right with the corresponding p-value.*

**2.** Page 4, Paragraph 1: To obtain the day-night genes, only gene expressions in the day time range [08:00-17:00) and the night time range [21:00-05:00) were compared. The interval choice seems too subjective, and even the length of the two intervals is not the same. Furthermore, the approach seems inefficient because, i.e., the twilight data (222 individuals) was not used. Importantly, this leads to the biased selection of rhythmic genes. Specifically, if the genes' rhythm has max at 19:00 or 7:00, the current approach cannot detect it as the day-night genes.

**Response:** We thank the Reviewer for bringing up this important consideration. We would like to clarify that these decisions are by design and explain the reasons behind them. As mentioned in the manuscript, given the difficulties to study rhythmicity using standard methodologies, we decided to explicitly focus on day vs. night variation. While we expect a high overlap between the two types of variation (day-night and circadian), it is important to keep in mind that they are not exactly the same. To investigate day-night differences, we therefore selected time intervals for which day or night can be confidently called (based on USA day-night timelines along the year). For this reason, the two intervals do not have the same length (there are more light hours than dark hours) and, also, we discard the twilight, which, in fact, should be better regarded as "uncertain data points" for our specific purpose. We have now stated these considerations more clearly in the revised manuscript.

Here is an alternative approach. Let's set up two moving windows such as [i, i+9] and [i+11, i+19] for i=0,1,2,3,… and then identify genes having different expressions between two intervals for any choice of i. In this way, a larger number of day-night genes can be identified.

**Response:** Although, as explained above, our explicit purpose was to study day-night differences (and therefore we disregarded twilight/uncertain genes, as acknowledged in the manuscript), we have implemented a sliding window approach, following the Reviewer's suggestion. Specifically, we identified the genes differentially expressed between two 6-hour windows, one between [i, i+6) for "day" and the second between [i+12, i+18) for "night" (note that we have not used the exact intervals proposed by the Reviewer since they were also not of equal length and, we think, the ones we chose could represent better circadian variation). When aggregating across all tissues, after correcting p-value per gene per tissue for the 12 windows, the largest numbers of differentially expressed genes in the two-window comparisons were obtained with i=7 and i=8, i.e. 7/8AM-1/2PM vs 7/8PM-1/2AM (new Supplemental Fig. S3A), which is consistent with our definition of day-night intervals windows, despite the multiple methodological differences. This supports, in our opinion, that day-night variation is a major contribution to circadian variation in gene expression, as expected. Moreover, and more importantly, we overall found a good correspondence between the number of genes differentially expressed in at least one window comparison (circadian-like genes) and the number of day-night genes per tissue (Figure R3 and new Supplemental Fig. S3B), despite the fairly different approach. We have included these results and the corresponding supplemental figure in the revised version of our manuscript.

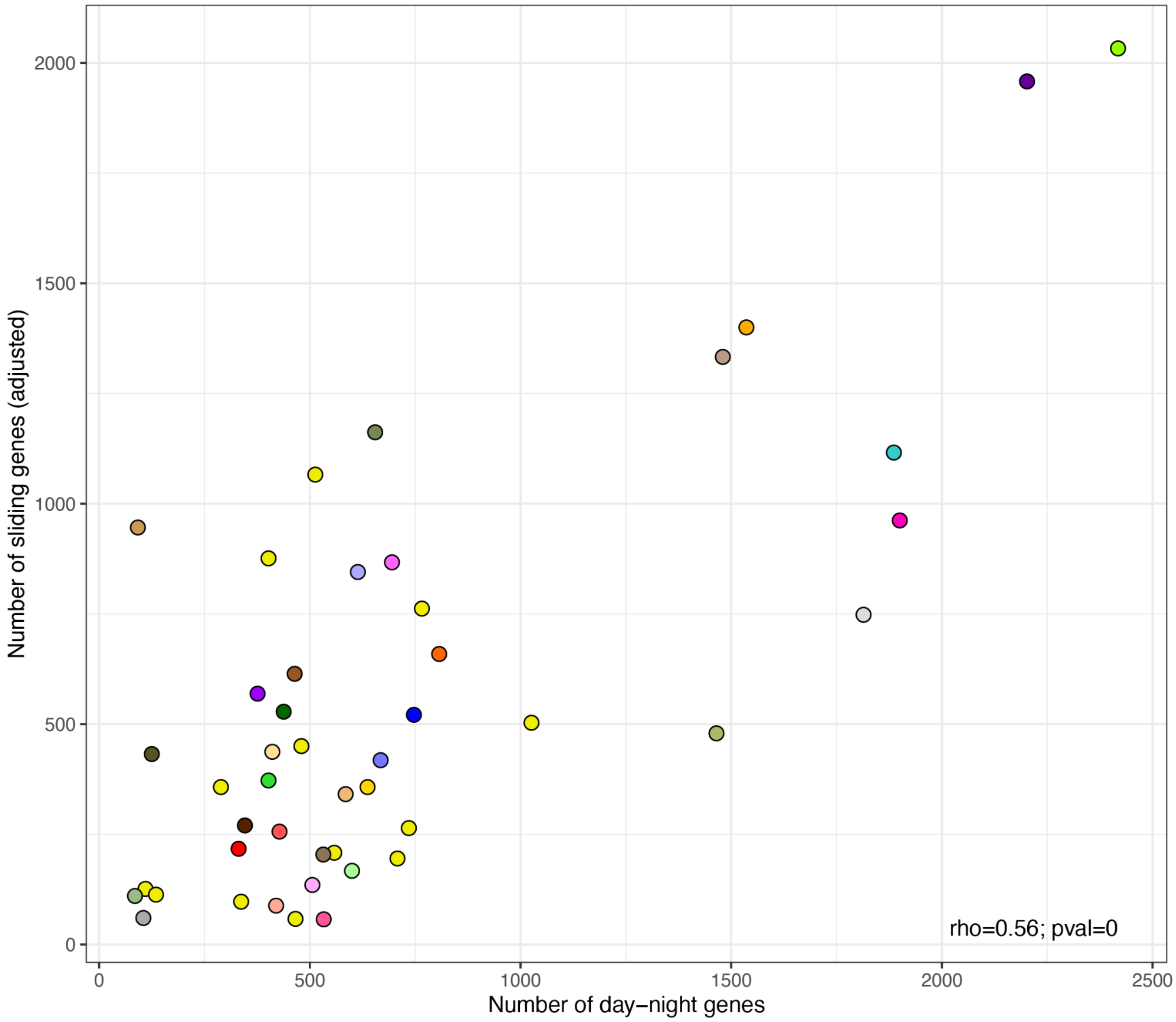


***Figure R3***: *Number of genes detected by the sliding window approach (Y axis) vs. our day-night approach (X axis). The Spearman rho is added on the bottom-right with the corresponding p-value.*

**3.** By comparing the number of rhythmic genes, the author evaluates the similarity between a baboon and human day-night genes (4p2p7l) or between seasonal genes and day-night genes (8p2p). However, comparing the number of genes is too simplistic to evaluate the similarity between the two groups. Instead, please use the Jaccard index; you can more accurately check the similarity between two groups of rhythmic genes.

**Response:** We thank the Reviewer for their comment and their suggestion regarding the Jaccard index. We would like to clarify that we were not comparing those numbers to claim that the patterns were similar between human and baboon (or day-night and seasons, which is in fact the opposite). We simply meant to mention that the overall numbers were comparable. Then, for the human-baboon comparison, we only focused on core clock genes, and for day-night vs seasons we performed more detailed analyses (e.g., direct comparisons by tissues, etc.).

Nonetheless, we fully agree this is a nice suggestion. We have thus computed the overall Jaccard index as well as the Simpson index (similar to the Jaccard index but dividing the size of the intersection by the size of the smallest set) between human and baboon, using only the

genes having a homologue in the other species and the similar tissues (see Supplemental Table S2). We obtained a Jaccard index of 0.46 and a Simpson index of 0.7. For day-night and seasonal genes, we have performed this analysis for our high-confidence sets, obtaining a Jaccard index of 0.058 and a Simpson index of 0.27. These values have now been included in the manuscript.

**4.** Page 8, Paragraph 1: The number/fraction of seasonal genes is much higher than those of day-night genes. However, this result seems biased due to the different analysis methods used to detect seasonal and day-night genes. For the seasonal genes, if the expression of a gene of the four windows across the season is distinguished, it is considered a seasonal gene. On the other hand, for day-night genes, two windows are used. Of course, the chance that one of four is distinguished is higher than one of two. Please perform a consistent analysis. Specifically, the moving window approach with the two windows, suggested above, can be used for the seasonal gene detection to test whether the still the number of seasonal genes are higher than the number of day-night genes and there is no correlation between them across tissues.

**Response:** We would like to note that for each season we make only one comparison: that season against the other three. We believe that this is the most comparable to our day-night comparison. However, we agree that when the four seasons are pooled together, the total number of genes identified increases. For this reason, we did not intend to make any relevant claim about the actual number of genes in each type of variation (as also explained in the previous point); we focused on the relative comparisons within and among tissues and the levels of gene overlap, which are fair with respect to the considerations raised by the Reviewer. We have now modified this statement in the Results section and acknowledge the bias with regards to the absolute numbers. We thank the Reviewer for bringing this up and apologize for the confusion.

With regards to the suggestion of applying a sliding window strategy, we unfortunately do not have information of the day, or even the month, of death. Therefore, a sliding window approach is not feasible here, as it would only allow for two comparisons (spring/summer vs fall/winter and summer/fall against winter/spring). Given the limitations of the GTEx data, we reason that our approach is the most robust to the fact that only the season of death is available to us. To give an example, two individuals could have died one day apart but in two different seasons, or 90 days apart but still within the same season. By doing one season against the rest, we believe the boundary effect is minimized.

**5.** Page 12 Discussion: In the previous study, the time of death was inferred from the expression of known circadian genes. On the other hand, in this study, the actual death time is used. Please investigate how such times are different for each death case (highly correlated?). If they are not highly correlated, please justify the choice of the time.

**Response:** This issue was also raised by Reviewer #1 (Point #1). In short, we observed little correlation between the CYCLOPS inferred time of death and the GTEx reported time of death. Importantly, while GTEx reported times are evenly distributed across the day-night cycle, most times inferred by CYCLOPS are at phase $0 = 2\pi$ (Figure R4). Moreover, in some cases, CYCLOPS infers different times for different tissues within the same individuals. For these reasons, we prefer not to include these results in our manuscript.

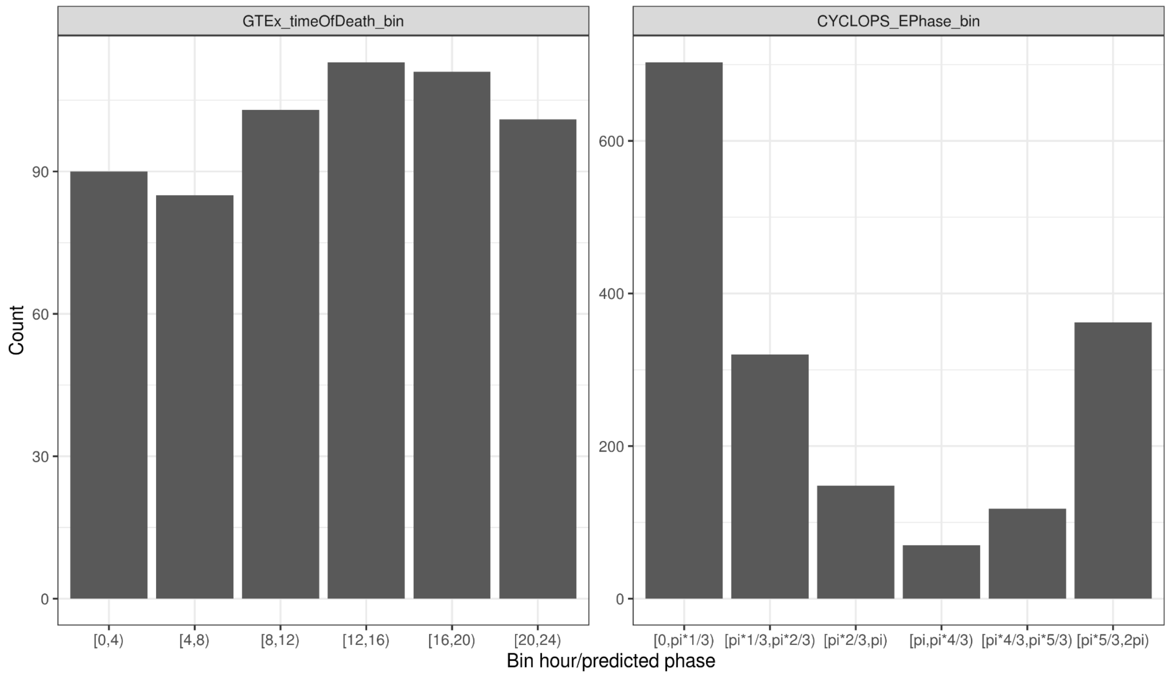


***Figure R4:*** *Distribution of GTEx samples based on the reported time of death (left) and the estimated time by CYCLOPS (right).*

**Minor**:

**6.** Page 7 Paragraph 2: 254 genes known to be related to insomnia are considered. Are there any genes related to circadian rhythms sleep disorders?

**Response**: Thank you for the suggestion. From the four intrinsic circadian rhythm sleep disorders, only "delayed sleep phase disorder" and "advanced sleep phase disorder" were present in MalaCards (www.malacards.org) and had 34 and 25 genes associated, respectively (38 unique genes in total). Of these 38 genes, 11 are in our high-confidence day-night genes set (11 advanced and 10 delayed sleep phase syndrome), of which 10 are part of the core clock: *ARNTL*, *CLOCK*, *CRY1*, *CRY2*, *DBP*, *NPAS2*, *NR1D1*, *NR1D2*, *PER2*, *PER3*, with the exception of *CATSPER2*. We have added a sentence reporting this result in the manuscript.

**7.** Page 11 Paragraph 2: The authors analyzed seasonal differences in cell-type-specific markers in the brain. In a recent study (Shao'ang Wen, Nat. Neuroscience, 2020), key marker genes of sub-populations of SCN have been reported. Please discuss whether those marker genes are included in the identified day-night genes/seasonal genes in the brain.

**Response**: We have added an additional figure (Supplemental Fig. S20) analyzing the differential expression of these markers across brain regions and seasons. Overall, these results are highly consistent with those we reported in Fig. 6. We have decided not to include the results regarding day-night variation (0/627 markers are in our high-confidence day-night set) since GTEx does not include data for SCN, which would be the most relevant brain region.

**8.** It is well known that the photo-period varies depending on the season, affecting the core clock genes of the Suprachiasmatic nucleus (SCN), the "master clock" of mammals. (Evans et al. BMC Biology, 2015, Gerald Lincoln, PNAS, 2002, Z. BENDOVÁ, Physiol. Res, 2006). It

would be interesting to check whether expression levels of day-night genes change depending on the season. In particular, please check whether the expression of the core clock genes shows any seasonality.

**Response**: In our original submission, we included a table (Supplemental Table S12) in which we provide the number of day-night and seasonal genes per tissue and the intersection. Following the suggestion by the Reviewer, in this revised version we further include an additional table with the tissue and season in which core clock and other high-confidence day-night genes are seasonal (Supplemental Table S15). Specifically, among the 16 core clock genes, three are included in our high-confidence seasonal gene set: *PER3* (fall, summer, winter), *PER1* (winter), *NR1D2* (fall).

**9.** Page 20: Why are the numbers of the marker genes for microglia (9) and neuron (11) set differently from the numbers of other marker genes (10)?

R: We originally selected the same number of markers for each cell type, but not all had sufficient expression and were protein-coding in our dataset.

**10.** Discussion: When a drug targets molecules whose level changes in a circadian manner, the efficacy of the drug can be changed depending on the dosing time of day (i.e., chronotherapy) (https://doi.org/10.1038/s41574-018-0142-x, https://doi.org/10.15252/msb.20198838). Similarly, when a drug targets seasonal genes, we can expect seasonality in drug efficacy. It would be great to discuss whether the identified day-night genes and seasonal genes in this study are targets of drugs, as done in Zhang et al. PNAS (ref 1 of the manuscript).

**Response**: We thank the Reviewer for this great suggestion, which, we believe, adds interest to our manuscript and resource. We have used the Drug-Gene Interaction database (DGI, www.dgidb.org), which contains gene targets for about 11,049 drugs. First, we found that 91 of the 445 (20%) high-confidence day-night genes are targeted by a total of 1,077 drugs (Supplemental Tables S18-S20). Second, we observed that 307 high-confidence seasonal genes (18%) are targeted by 2,632 drugs present in DGI (Supplemental Tables S21-S23). Interestingly, for the 18 drugs targeting at least 10 high-confidence seasonal genes, we observed 11 anti-cancer drugs, including bortezomib (16 targets), cisplatin (15), sorafenib (12), doxorubicin (12), and ME-344 (12) (in clinical trial). Therefore, our findings could provide novel insights into the potential interactions between some drug treatments and seasonality for specific tissues, which could eventually help clinicians adjust doses and treatments. We have added a new paragraph reporting these data in the Results section of the manuscript, provided new supplemental tables detailing all the information, and added a mention in the Discussion section.
